# Reconstructed Copper Compound by Cerium Leaching for Enhanced Electrochemical CO_2_‐to‐Ethylene Conversion

**DOI:** 10.1002/advs.202504094

**Published:** 2026-01-02

**Authors:** Jinxian Feng, Chunfa Liu, Yu‐Xuan Xiao, Lun Li, Keyu An, Sen Ding, Weng Fai Ip, Daniel H. C. Chua, Hui Pan

**Affiliations:** ^1^ Institute of Applied Physics and Materials Engineering University of Macau Macao SAR 999078 China; ^2^ Department of Physics and Chemistry Faculty of Science and Technology University of Macau Macao SAR 999078 China; ^3^ Department of Materials Science and Engineering National University of Singapore Singapore City 119077 Singapore

**Keywords:** CO_2_ reduction, Cu compounds, ethylene, leaching, reconstruction

## Abstract

Rationally modulating the reconstructed structure of electrocatalyst is an effective avenue toward selective electrochemical CO_2_ reduction (e‐CO_2_RR). Herein, it is reported that a Ce‐incorporated Cu oxide (CeCuO_x_) can have prominent performance for the selective production of C_2_H_4_ with a high Faraday efficiency (FE) of 55.39% at −0.93 V, and partial current density of −39.50 mA cm^−2^ at −1.03 V, higher than those of Cu oxide and Cu at the same potential. Systematical experimental investigations show that the CeCuO_x_ reconstructs to oxygen‐contained Cu with optimized Cu^0^/(Cu^+^+Cu^2+^) ratio and electronic structure through Ce leaching and in situ reduction. All of those benefit water molecule cleavage and OH^−^ accumulation that could provide hydrogen feedstock and suppress competitive hydrogen evolution. Importantly, specific C_1–2_ intermediates generation and activation are enhanced, achieving high C_2_H_4_ selectivity and efficiency. Our work provides new insights into the relationship between the reconstructed structure and mechanism for boosting CO_2_ to multi‐carbon products.

## Graphical abstract

A Ce‐incorporated Cu oxide can achieve prominent selective CO_2_‐to‐ethylene performance by reconstructing to oxygen‐contained Cu with an optimized Cu^0^/(Cu^+^+Cu^2+^) ratio and electronic structure through Ce leaching and in situ reduction. The interactions between the reconstructed structure and leached Ce improve the CO_2_‐to‐ethylene by enhancing water molecule cleavage, suppressing hydrogen evolution and optimizing carbon reaction pathways.

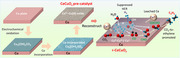

## 1 Introduction

Driven by sustainable electricity, converting carbon dioxide (CO_2_) to value‐added multi‐carbon products via electrochemical CO_2_ reduction reaction (e‐CO_2_RR) offers a promising way to mitigate CO_2_ emission and make the renewable upgrading of CO_2_.^[^ ^1^ ^]^ As an important multi‐carbon organic molecule, ethylene has been widely used in the chemical industry^[^ ^2^ ^]^ and is one of important intermediates for fuel synthesis.^[^ ^3^ ^]^ Copper (Cu) and its alloys, oxides, chalcogenides, and metal‐organic compounds, have been commonly recognized as main electrocatalysts that could convert CO_2_ to ethylene due to their crystal and electronic structure flexibility.^[^ ^4^ ^]^ However, Cu‐based catalysts typically give these disadvantages that make electrocatalytic CO_2_‐to‐ethylene conversion far from practical:^[^ ^5^ ^]^ i) serious competitive hydrogen evolution reaction (HER), ii) high kinetic obstacle and iii) poor stability.

For decades, it has been proved that the surface valence state, crystalline and chemical composition of as‐prepared material for electrocatalysis (called pre‐catalyst) may be reconstructed in the e‐CO_2_RR process,^[^ ^6^ ^]^ which is determined by pre‐catalyst's structure and electrocatalytic condition. The reconstruction may destroy the catalytic structure, but it may also create defects, distortions, interfaces, etc., which play “vital few” effect on electrocatalysis.^[^ ^4a^ ^]^ For the Cu‐based electrocatalysts for CO_2_‐to‐ethylene conversion, the Cu^0^ is regarded as CO_2_‐to‐C_1_ intermediates (CO, COOH, CHO, etc.) conversion site, while the Cu^+^/Cu^2+^ sites are regarded as sites for C‐C coupling through capture C_1_ intermediates.^[^ ^7^ ^]^ However, the reconstruction may alter the ratio and electronic structures of the Cu^0^, Cu^+^, and Cu^2+^ in the catalysts that bring unpredictable electrochemical performance change in CO_2_‐to‐ethylene conversion. Leaching may introduce distortion, defect and new phases that endows special electronic structure,^[^ ^8^ ^]^ and the interactions between the leached species and the reconstructed surface may also play role in the electrocatalysis.^[^ ^9^ ^]^ Unfortunately, the reconstructed fine structure by leaching and how it improves the e‐CO_2_RR kinetics are still need to be further studied.

**Figure 1:**
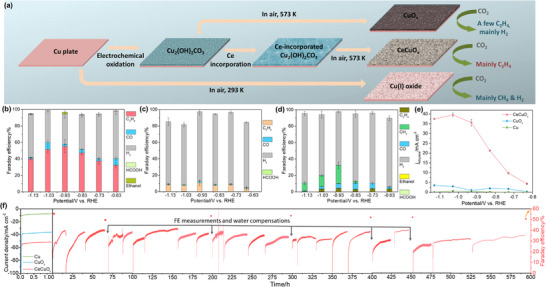
a) Scheme of pre‐catalyst fabrication and surface reconstruction. Distributions of products on: b) CeCuO_x_, c) CuO_x_ and d) Cu. (e) j_ethylene_. (f) A 590‐hour stability measurement with on‐and‐off galvanostatic tests at ‐0.93 V.

In this work, we fabricate Ce‐incorporated Cu oxide (CeCuO_x_) as a pre‐electrocatalyst based on Cu plate electrooxidation, element penetration and annealing (**Figure** **1**a). The CeCuO_x_ shows maximum ethylene Faraday efficiency (FE) of 55.39% at −0.93 V and yields maximum ethylene partial current density (j_ethylene_) of −39.50 mA cm^−2^ at −1.03 V, as well as excellent long‐term stability up to 590 h (< 5% ethylene FE loss). We identify that Ce is leached in electrocatalysis and CeCuO_x_ reconstructs to oxygen‐contained Cu on surface. Notably, the Ce‐leaching regulates the Cu^0^/(Cu^+^+Cu^2+^) ratio of reconstructed oxygen‐contained Cu and optimizes the electronic state, which triggers high Cu (100) ratio in the electrocatalysis. Therefore, the CO_2_‐to‐ethylene kinetics is promoted by: i) enhancing the water cleavage and forming active hydrogen species for the hydrogenation and deoxygenation of CO_2_ and intermediates, ii) providing OH^−^ affinity surface that prevents competitive HER, and iii) promoting specific C_1∼2_ intermediates generation and activation for the ethylene production.

## 2 Results and Discussion

### 2.1 Structural Characterizations of Pre‐Electrocatalyst

CeCuO_x_ is fabricated as pre‐catalyst based on commercial Cu plate (supporting information). Briefly, the Cu is first electrooxidized to Cu_2_(OH)_2_CO_3_ and converted to Ce‐incorporated Cu_2_(OH)_2_CO_3_ in the following Ce incorporation process (Figure S1, Supporting Information). The CeCuO_x_ is eventually obtained after annealing in the air. The Cu oxide (CuO_x_) and Cu plate after surface cleaning (Cu) are used for references. Scanning electronic microscopy (SEM) images show that CeCuO_x_, CuO_x_ and Cu are consisted by nanoparticles with≈100, 50 and 20 nm in sizes, respectively (Figures S2a,b, S3a,b, and S4a,b, Supporting Information), and the element disperse spectroscopy (EDS) shows the co‐existence of Cu, Ce, and O signals in CeCuO_x_ (Figure S2c–e, Supporting Information), while the CuO_x_ and Cu show Cu and O signal only (Figures S3c–f and S4c–f, Supporting Information). The X‐ray diffraction (XRD) shows the Cu and Cu_2_O characteristic peaks in the diffraction pattern of Cu pre‐catalyst (Figure S5a, Supporting Information), while the CuO_x_ and CeCuO_x_ show the Cu, Cu_2_O, and CuO diffraction peaks (Figure S5b, Supporting Information). Notably, the diffraction peak of Ce hardly be observed in the XRD pattern of CeCuO_x_. Meanwhile, the diffraction peaks of CuO and Cu_2_O have obvious shifts (Figure S5b, Supporting Information), indicating the Ce incorporation. The Raman spectra of CeCuO_x_, CuO_x_, and Cu show the peaks at ≈216 and 264 cm^−1^, corresponding to the Cu‐O vibrations in Cu_2_O, while the peaks at 450 and 632 cm^−1^ related to the Cu‐O vibrations in CuO.^[^ ^10^ ^]^ Specially, for CeCuO_x_, the concurrent observation of Ce‐O peak confirms the successful synthesis of the Ce‐incorporated Cu oxide further (Figure S5c, Supporting Information).^[^ ^11^ ^]^ The X‐ray photoelectron spectroscopy (XPS) spectra for O 1s show the metal‐O, metal‐OH and chemically adsorbed water molecules exist in all the samples (Figure S6a, Supporting Information).^[^ ^12^ ^]^ For the CeCuO_x_, the XPS Ce 3d spectrum exhibits Ce^3+^ and Ce^4+^ (Figure S6b, Supporting Information),^[^ ^13^ ^]^ indicating the Ce incorporation further. The XPS Cu 2p_3/2_ and 2p_1/2_, as well as their satellite peaks show the existences of CuO in CeCuO_x_ and CuO_x_, while Cu or Cu_2_O in Cu (Figure S6c, Supporting Information).^[^ ^13b^ ^]^ The Cu LMM spectra show the co‐existence of Cu^2+^, Cu^+^, and Cu^0^ on CuO_x_, Cu and CeCuO_x_ surfaces (Figure S6d, Supporting Information).^[^ ^14^ ^]^ All those results suggest that CeCuO_x_ is composed of Ce‐incorporated CuO, and CuO_x_ is dominated by CuO, which contains Cu^+^ and Cu^0^ (Figure S6d, Supporting Information), while the Cu is composed of Cu_2_O with Cu^0^ and Cu^2+^ (Figure S6d, Supporting Information). Notably, the binding energy of Cu^2+^ follows the trend: CuO_x_ ≈ CeCuO_x_ > Cu, while the binding energy of Cu^+^ and Cu^0^ follow the trend: CuO_x_ > CeCuO_x_ > Cu (Figure S6d, Supporting Information), suggesting the electronic structures of the pre‐catalysts are different, which shall be the key to in situ reconstruction that affects the electrochemical performance.

### 2.2 e‐CO_2_RR Performances

The e‐CO_2_RR performance was measured in CO_2_‐saturated 0.5 m KHCO_3_, and the liquid and gaseous products were examined by high‐performance liquid chronography (HPLC) and on‐line gas chronography (GC), respectively. The potentiostatic electrolysis experiments were performed in the range of −0.63–−1.13 V. For the CeCuO_x_, gaseous products, including ethylene, CO and H_2_, as well as liquid products, including HCOO^−^ and ethanol, can be observed (Figures S7a and S8a, Supporting Information). Its maximum ethylene FE is up to 55.39% at −0.93 V, and the maximum j_ethylene_ is −39.50 mA cm^−2^ at −1.03 V. At −0.93 V, hydrogen is the main by‐product (FE = 36.38%), together with minor CO (FE = 2.47%), ethanol (FE = 1.94%) and HCOO^−^ (FE = 0.76%) (Figure 1b). Notably, the ethylene FEs of CeCuO_x_ are higher than 40% in a voltage range of from −0.83 to −1.13 V (Figure 1b). As a comparison, CuO_x_ can catalyze gaseous products including ethylene, CO and H_2_, as well as liquid product, HCOO^−^, in e‐CO_2_RR (Figures S7b and S8b, Supporting Information). Its maximum ethylene FE is only 10.81% at −0.93 V, and the maximum j_ethylene_ is −3.55 mA cm^−2^ at −1.13 V. Besides, hydrogen (FE = 84.92%) and CO (FE = 1.71%) as well as HCOO^−^ (FE = 0.07%) are also detected at ‐0.93 V as by‐products (Figure 1c). The Cu catalyzes gaseous products like ethylene, methane, CO and H_2_, and liquid products including HCOO^−^ and ethanol (Figures S7c and S8c, Supporting Information). Its maximum ethylene FE is only 4.23% at −0.93 V, and the maximum j_ethylene_ is −0.61 mA cm^−2^ at −1.03 V. At −0.93 V, hydrogen (FE = 50.62%), methane (FE = 22.32%), CO (FE = 5.92%), HCOO^−^ (FE = 0.76%) and ethanol (FE = 0.12%) are also detected, indicating the CO_2_ undergoes deep reduction to methane mainly (Figure 1d). Moreover, CeCuO_x_ shows higher turnover frequency (TOF) than those of CuO_x_ and Cu at the same potential (Table S1 and Figure S9, Supporting Information). Therefore, the Ce incorporation endows high ethylene selectively, j_ethylene_ and TOF of electrocatalyst (Figure 1b–e). Additionally, when the Ce is incorporated into Cu oxide using different Ce(NO_3_)_3_ concentrations, the FE ethylene and j_ethylene_ are also higher than those of CuO_x_ and Cu (Figure S10, Supporting Information). Also, the La^3+^‐incorporated CuO_x_ (LaCuO_x_) shows maximal ethylene FEs of 19.0% at −1.13 V, and the Al^3+^‐incorporated CuO_x_ (AlCuO_x_) shows maximal ethylene FEs 27.4% at −1.03 V (Figures S11 and S12, Supporting Information). The LaCuO_x_ achieves maximal j_ethylene_ −11.65 mA cm^−2^ at −1.13 V, and the AlCuO_x_ achieves maximal j_ethylene_ −20.37 mA cm^−2^ at −1.03 V (Figure S12, Supporting Information). Both the ethylene FE and j_ethylene_ of ion‐incorporated samples are higher than those of CuO_x_ and Cu, too, indicating the universal of the +3‐transition element incorporation strategy. Notably, the CeCuO_x_ demonstrates a competitive CO_2_RR performance compared with the literature under the same conditions (Table S2, Supporting Information). Importantly, the CeCuO_x_ shows an excellent long‐term stability test at −0.93 V for ∼590 h on‐and‐off accelerate degradation test with negligible FE decreasing (< 5% FE ethylene loss) (Figure 1f).

### 2.3 Structural Evolution

**Figure 2:**
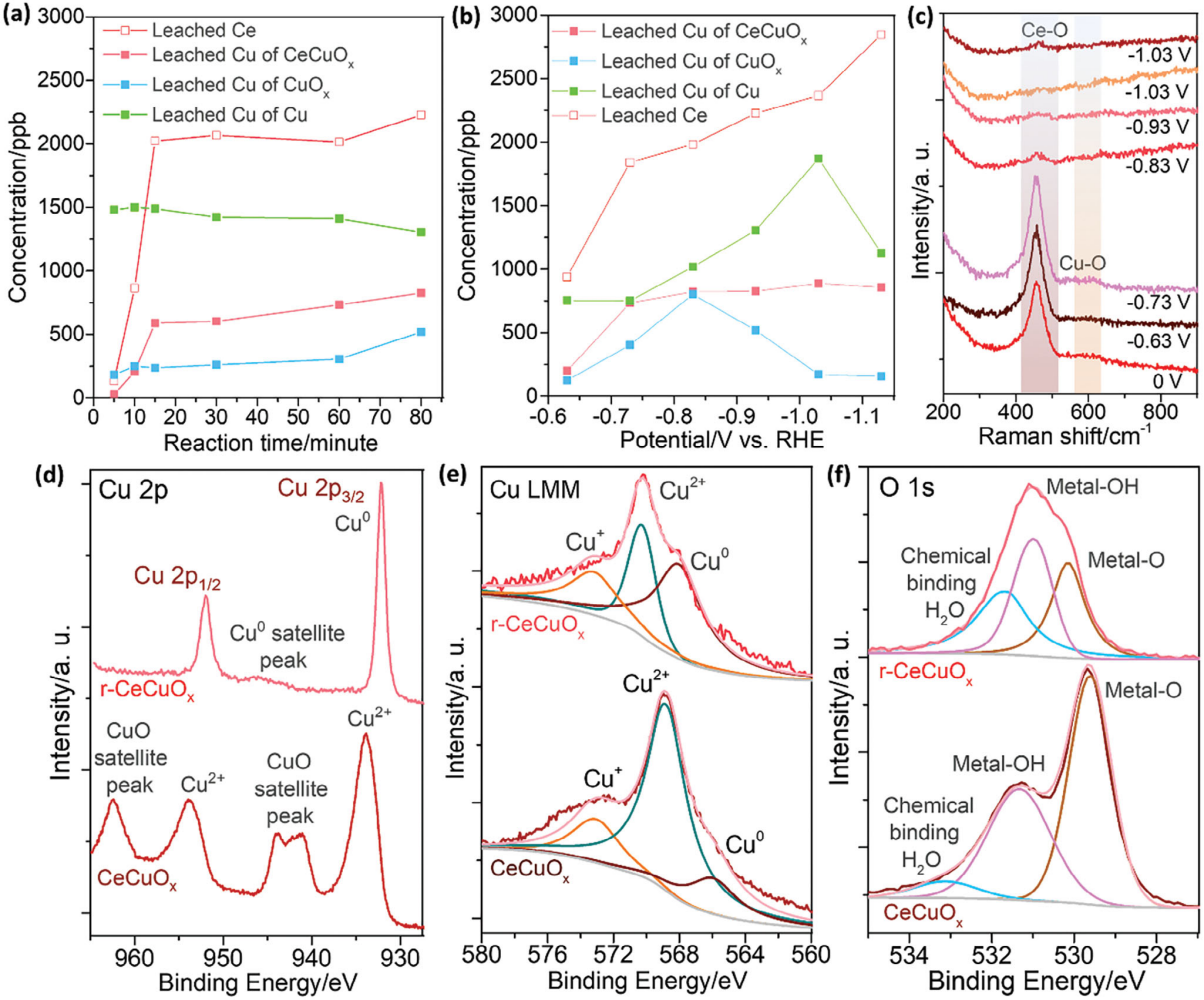
a) Reaction time‐dependent concentrations of leached ions for CeCuO_x_, CuO_x_, and Cu measured at ‐0.93 V, and b) potential‐dependent concentrations of leached ions for CeCuO_x_, CuO_x_, and Cu measured at the 80th min. c) In‐situ Raman spectra of CeCuO_x_ in e‐CO_2_RR at different potentials. d) XPS Cu 2p, e) Cu LMM and f) XPS O 1s spectra of CeCuO_x_ before and after tests.

To elucidate the dynamic changes of the surface structure of Cu, CuO_x_, and CeCuO_x_ in the catalysis, systematical characterizations were held. The inductively coupled plasma‐mass spectrometry (ICP‐MS) shows that the concentrations of Ce and Cu increase sharply with potential increased initially and keep steady after 30 minutes at −0.93 V (**Figure** **2**a,b). However, the Ce can be detected in the CeCuO_x_ after 70‐min test, suggesting the Ce dissolution shall only happen on the surface (Table S2, Supporting Information). Additionally, the Cu can also be dissolved, too, but the Ce concentration is much higher than that of Cu, suggesting the Ce is more prone to being leached (Figure 2a,b).

To study the surface chemical states with the potential change, we established the potential dependent in‐situ Raman spectroscopy. It is shown that the Ce‐O characteristic peak diminishes gradually, and vanishes at the potential over −0.83 V, indicating the Ce on surface is leached completely when the potential is negative than −0.83 V (Figure 2c). The in‐situ Raman spectra measured from 0th to 30th min at −0.93 V show that the Ce‐O signal diminishes and vanishes after 15th min, indicating the Ce on surface is completely leached (Figure S13, Supporting Information), aligned well with the ICP‐MS results (Table S3, Supporting Information). Additionally, the Cu‐O characteristic peaks for all the samples diminish gradually and finally vanish when potential increases from −0.63 to −1.13 V, indicating the Cu metal phase should be the active site at the potential of maximum FE (Figure 2c; Figure S14, Supporting Information). The XRD patterns also show the intensities of CuO and Cu_2_O diffraction peaks for CeCuO_x_, CuO_x_ and Cu after 70‐min test (denotes as r‐CeCuO_x_, r‐CuO_x_, and r‐Cu, respectively) are lower than those of pre‐catalysts, suggesting the reduction of Cu oxides. Notably, we can see that the diffraction peaks of Cu(OH)_2_ appear in the XRD patterns of r‐CeCuO_x_ and r‐CuO_x_, suggesting the hydration occurs (Figure S15, Supporting Information).

XPS was used to study the chemical state change before and after electrocatalysis. The XPS Ce 3d spectrum of r‐CeCuO_x_ shows the Ce signal disappears, indicating the Ce is leached (Figure S16a, Supporting Information). For CeCuO_x_ and Cu, the Cu 2p_3/2_, 2p_1/2_ and satellite peaks in their XPS Cu 2p spectra show the CuO converts to Cu metal (Figure 2d; Figure S16b, Supporting Information), while the CuO in CuO_x_ converts to Cu_2_O (Figure S16d, Supporting Information). The Cu LMM Auger spectra show the co‐existence of Cu^0^, Cu^+^ and Cu^2+^ for all the sample after electrocatalysis (Figure 2e; Figure S16c,e, Supporting Information).^[^ ^9^, ^15^ ^]^ Additionally, for CeCuO_x_ and CuO_x_, their O 1s spectra show the ratios of metal‐OH and chemical bonded water characteristic peaks become higher after the 70‐min test, indicating the surface is hydrated in electrocatalysis (Figure 2f; Figure S16f, Supporting Information).^[^ ^12^, ^16^ ^]^ For Cu, the O 1s spectra show the ratios of metal‐OH and chemical bonded water characteristic peaks become lower after the 70‐minute test, indicating the surface become hydrophobic (Figure S16g, Supporting Information). Notably, the XPS spectra of r‐CeCuO_x_ after 590 h test shows negligible changes compared with those measured after 70‐min test (Figure S17, Supporting Information), indicating the stability of the reconstructed structure. Additionally, the electron spin resonance (ESR) spectra show that the unpaired electron peak intensity of r‐CeCuO_x_ increases more sharply than that of CeCuO_x_. On the contrary, ESR unpaired electron peak intensities of r‐CuO_x_ and r‐Cu show seldomly increasing than those of CuO_x_ and r‐Cu, respectively, indicating the Ce incorporation and leaching introduce more defects and vacancies (Figure S18, Supporting Information).

**Figure 3:**
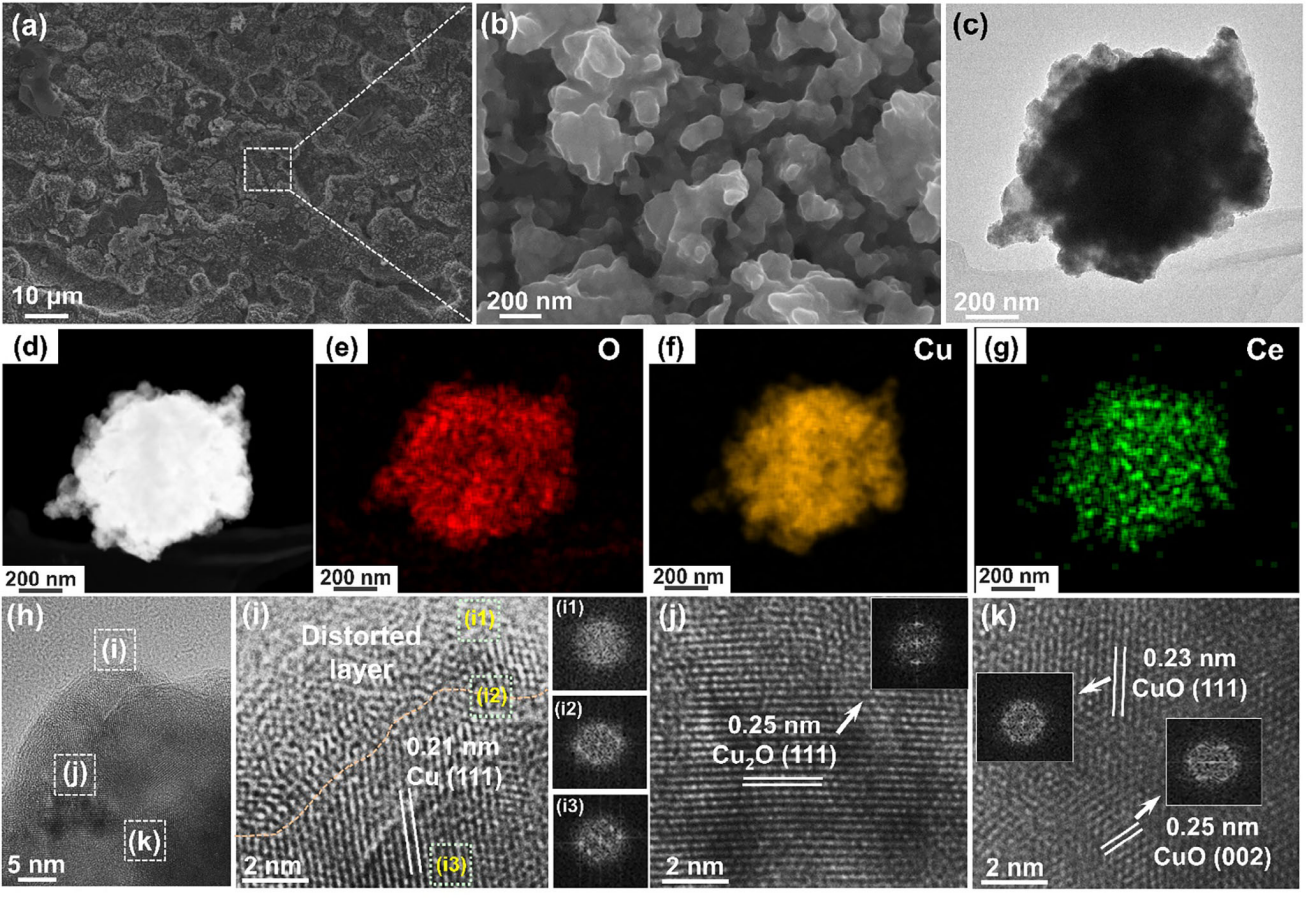
Morphology of r‐CeCuO_x_ after the 70‐minute e‐CO_2_RR test: a,b) SEM images.c) Low‐magnified TEM image. d) HAADF image and e–g) EDS mappings. (h) Magnified TEM image. Label: selected sites for HRTEM images in i–k). (i1‐i3): FFT patterns of i) from surface to inside.

To further elucidate the electrocatalyst's structure after reaction, systematical characterizations were carried out. The Cu and O of all the samples are uniformly distributed, while the Ce signal of r‐CeCuO_x_ is lower than the detection limit of EDS (~0.1% atomic ratio) (Figures S19–S21, Supporting Information). The SEM images show that the r‐CeCuO_x_ has a coral‐like network structure that is composed of small nanoparticles (**Figure** **3**a,b), and r‐CuO_x_ shows a dense nanoparticle aggregated network structure (Figure S20, Supporting Information), while the r‐Cu is composed of close‐packed nanoparticles (Figure S21, Supporting Information). The coral‐like structure of r‐CeCuO_x_ endows a lot of fissures that can expose abundant active sites that benefit for mass and charge transfer. The TEM image of r‐CeCuO_x_ shows that it is composed of various nanoparticles, and the EDS shows uniform distribution of Cu and O. Meanwhile, negligible Ce can be observed, confirming the Ce is leached further. The high‐resolution TEM (HRTEM) images were taken at different positions (labeled as i‐k in Figure 3h) to illustrate the fine structure from interface to bulk. The FFT of the corresponding HRTEM images show that the diffraction patterns from blurred to clear at position i1‐i3 zones in the blank i of Figure 3h, suggesting the poor crystalline structure to well crystalline structure from surface to inside. At position i, the spacing of lattice fringe is 0.21 nm. Combining with the FFT pattern, we may infer that it should correspond to Cu (111) (Figure 3i). The lattice fringe at position j shows a spacing of 0.25 nm, which can be assigned to Cu_2_O (111) and is consistent with the FFT pattern (Figure 3j). There are two lattice spacings that can be observed in position k, 0.23 and 0.25 nm. Combining with the FFT patterns, they shall correspond to CuO (111) and CuO (002), respectively (Figure 3k). For the r‐CuO_x_ and r‐Cu, a homogeneous Cu and O distribution can be observed (Figures S22a–d, l–o, Supporting Information). The r‐CuO_x_ shows blurred FFT patterns and lattice fringes at all labelled positions, suggesting the amorphous structure (Figure S22e–h, Supporting Information). The r‐Cu shows lattice fringes with the spacings are all 0.21 nm (at positions n, o, p in Figure S22m, Supporting Information), which may be assigned to Cu (111) or Cu_2_O (111). Combining with the FFT patterns, we can infer that the species at the n and o positions are attributed to Cu (111), while that in p position is attributed to Cu_2_O (111) (Figure S22n–p, Supporting Information). The TEM images indicate that the r‐CeCuO_x_ has poor crystalline layer on surface with a few nanometers with crystalline Cu, Cu_2_O, and CuO inside, and the r‐CuO_x_ has poor crystallinity structure, while the r‐Cu is composed by Cu and Cu_2_O hybrid, which shall affect the CO_2_‐to‐ethylene performance.

**Figure 4:**
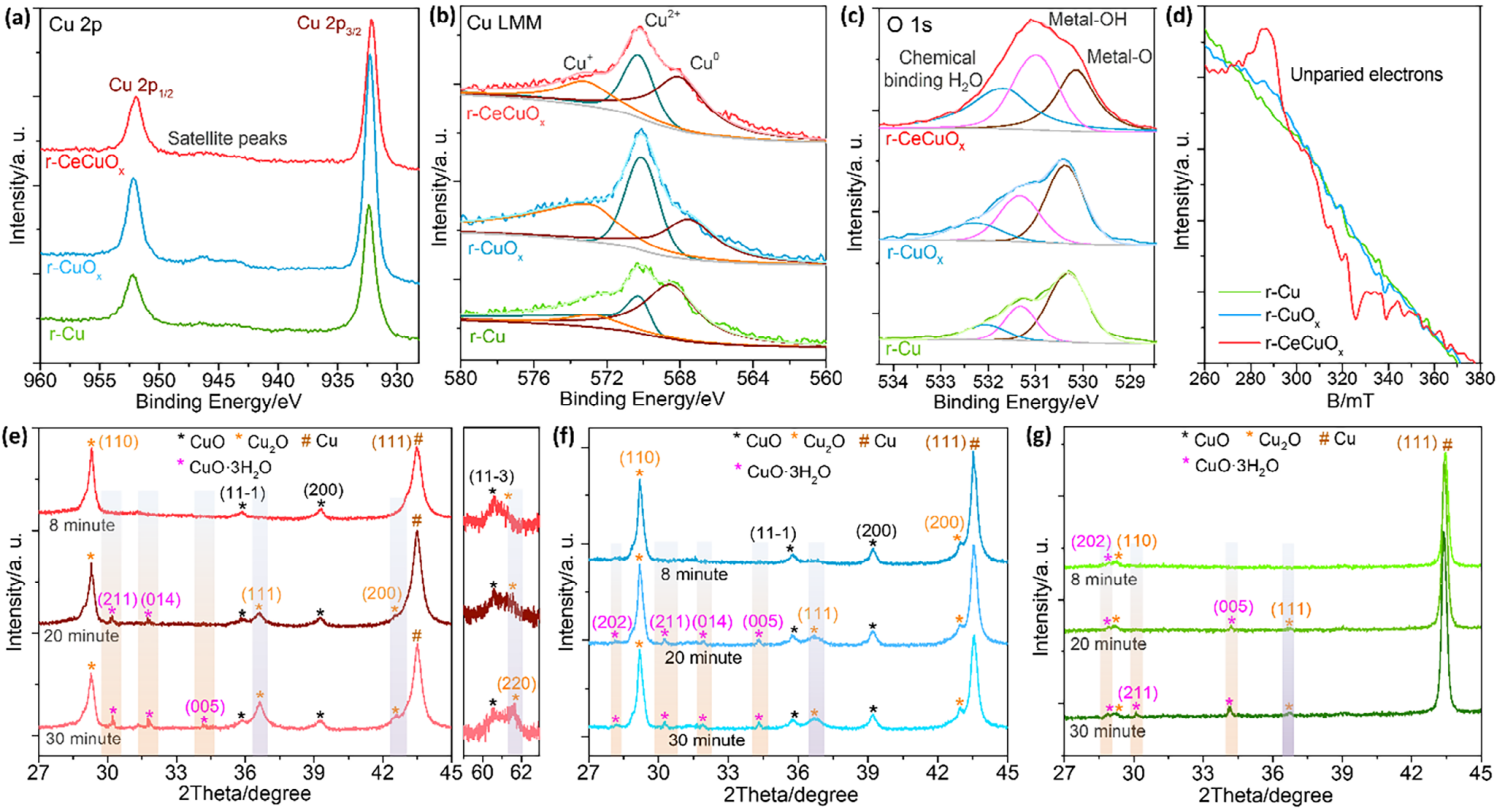
a) XPS Cu 2p, b) Cu LMM, and c) O 1s spectra after the 70‐min test. (d) Quasi in‐situ ESR spectra of r‐CeCuO_x_, r‐CuO_x,_ and r‐Cu. Quasi in situ XRD patterns of e) r‐CeCuO_x_, f) r‐CuO_x_ and g) r‐Cu.

XPS and quasi in situ ESR were utilized to study the chemical states after reaction. The XPS Cu 2p_3/2_ peaks and the features of the satellite peaks suggest the r‐CeCuO_x_ and r‐Cu surfaces are composed by metallic Cu mainly, while the r‐CuO_x_ surface is composed of Cu_2_O (**Figure** **4**a,b).^[^ ^14^, ^15^, ^17^ ^]^ Further, Cu LMM spectra show the co‐existence of Cu^0^, Cu^2+^, and Cu^+^ in all the samples.^[^ ^4a^ ^]^ The XPS O 1s spectra of all the samples show characteristic peaks of metal‐O, metal‐OH and chemically adsorbed water on all the samples, suggesting the structure of r‐CeCuO_x_ and r‐Cu is composed by metallic Cu with oxygen and chemical adsorption water, and the r‐CuO_x_ is composed by Cu_2_O with chemical adsorbed water. Notably, the ratio of Cu^0^ versus Cu^+^ and Cu^2+^ (Cu^0^/Cu^+^+Cu^2+^) follows the trend: r‐Cu > r‐CeCuO_x_ > r‐CuO_x_ (Table S4, Supporting Information), and the binding energy shifts in the Cu LMM spectra of all the samples after reaction show that the electron density of Cu^0^ in r‐CeCuO_x_ is higher than that in r‐Cu, but lower than that of r‐CuO_x_, while the Cu^+^ in r‐CeCuO_x_ has lower electron density than those in r‐CuO_x_ and r‐Cu (Table S5, Supporting Information). Meanwhile, the binding energies of metal‐O, metal‐OH and chemical binding water of r‐CeCuO_x_ show negative shift, indicating the O atoms of r‐CeCuO_x_ have higher electron density (Table S6, Supporting Information).^[^ ^18^ ^]^ Additionally, the quasi in situ ESR spectrum of r‐CeCuO_x_ after the 70‐min test shows stronger ESR peaks than that of r‐CuO_x_ and r‐Cu, suggesting more unpaired electrons (Figure 4d).^[^ ^6a^ ^]^ Those results indicate that the Ce leaching of CeCuO_x_ introduces Cu^2+^ and Cu^+^ ions with special surface electronic structures.

Quasi in‐situ XRD was monitored to study the reconstructed species. We used the in‐situ generated Cu oxides as probes to evaluate the activity of reconstructed Cu species. The quasi in‐situ XRD patterns were recorded on 8th, 20th, and 30th min after 70‐min test at −0.93 V. The XRD peaks of Cu_2_O (JCPDS #05‐0667) and CuO·3H_2_O (JCPDS #36‐0545) of r‐CeCuO_x_, r‐CuO_x_, and r‐Cu gradually present from 8th to 30th min, indicating the oxidization of reconstructed Cu (Figure 4e–g). Importantly, the rate of Cu(II) species (typically CuO·3H_2_O) generation of r‐CeCuO_x_ is less than that on r‐CuO_x_ and r‐Cu, indicating the reduced Cu species on r‐CeCuO_x_ is difficult to be oxidized and they are more stable (Figure 4e–g; Table S7, Supporting Information).^[^ ^19^ ^]^

Those results underscore that the Ce of CeCuO_x_ is leached and the CuO is reduced to oxygen‐contained Cu metal, while the Cu_2_O on Cu is reduced to oxygen‐contained Cu metal, too. The CuO on CuO_x_ surface is reduced to Cu_2_O in electrocatalysis. Notably, the electron distributions on Cu^0^, Cu^+^ and Cu^+^ are different, that may benefit different basic steps of e‐CO_2_RR that introduce different selectively.

### 2.4 Reaction Mechanism

**Figure 5:**
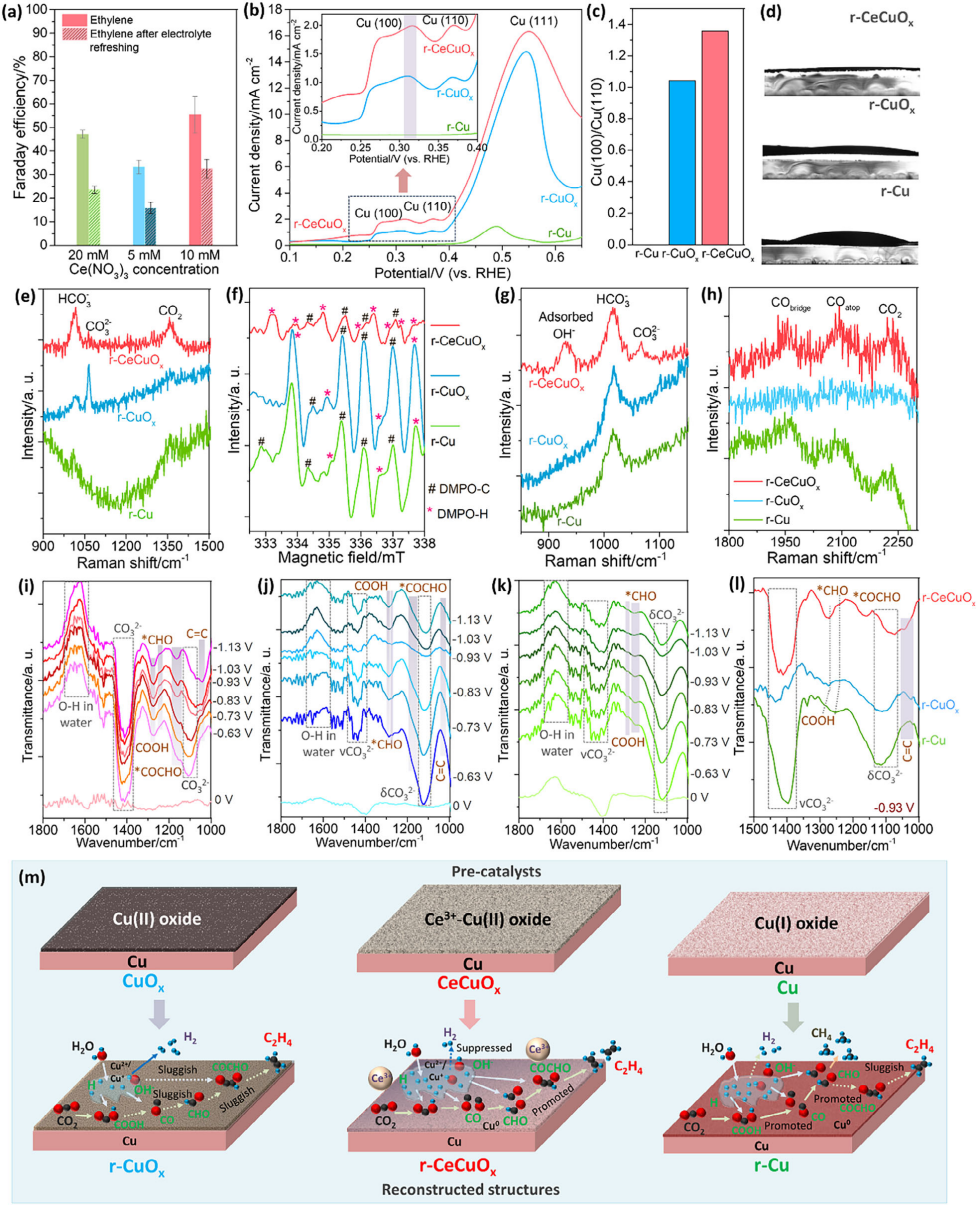
a) Ethylene FEs of CeCuO_x_ prepared by using different Ce(NO_3_)_3_ concentrations as Ce sources before and after electrolyte refreshing; b) CV curves measured in 1.0 m KOH; c) Cu (100)/Cu (110) ratios; d) Contact angle measurements; e) In situ Raman spectra measured within 900‐1500 cm^−1^ at ‐0.93 V; f) Quasi in‐situ ESR spectra; g) In situ Raman spectra measured at 30th second after the bias is terminated; h) In‐situ Raman spectra measured within 1800–2400 cm^−1^ of r‐Cu, r‐CuO_x_ and r‐CeCuO_x_ at −0.93 V. In‐situ FT‐IR ATR spectra measured within −0.63–−1.13 V: i) r‐CeCuO_x_; j) r‐CuO_x_ and k) r‐Cu. l) Comparison of in‐situ FT‐IR ATR spectra of r‐CeCuO_x_, r‐CuO_x_, and r‐Cu measured at −0.93 V. m) Scheme of reconstruction and e‐CO_2_RR pathway improvements.

The FE ratios between ethylene or methane and CO can be used to study the selectively of carbon reaction pathway. The FE ethylene/CO ratios of CeCuO_x_ are comparable to those of CuO_x_ at the potential > −0.83 V, but higher than those of CuO_x_ when the potential < −0.83 V (Figure S23a, Supporting Information). Therefore, the interactions between reconstructed Cu species and leached Ce for CeCuO_x_ improve the ethylene generation as the Ce begins to leach at −0.83 V (Figure 2c). For r‐Cu, the FE ethylene/CO ratios are smaller than those of CeCuO_x_, but the FE CH_4_/CO ratios significantly increase when the potential is negative than −0.83 V, indicating the CO_2_ is prone to generating CH_4_ (Figure S23b, Supporting Information). To further study the effect of leached Ce on the CO_2_‐to‐ethylene, another 70‐min test is held after the electrolyte is completely refreshed. We can see that the ethylene FEs decrease sharply, indicating the leached Ce improves the CO_2_‐to‐ethylene conversion (**Figure** **5**a). Nyquist plots of CeCuO_x_ prepared by different Ce(NO_3_)_3_ concentrations were measured after electrolyte refreshing. We can see that the radius of semicircles increase sharply, indicating the charge transfer process become sluggish (Figure S24, Supporting Information). Therefore, the leached Ce in the solution shall improve the CO_2_‐to‐ethylene conversion through charge transfer enhancement.

The electron and ion transfer properties can be studied by electrochemical impedance spectroscopy (EIS). Bode plots measured from −0.63 to −1.13 V for r‐CeCuO_x_, r‐CuO_x_ and r‐Cu show phase angles > −45°, indicating the Faraday process takes place mainly (Figure S25, Supporting Information). The Nyquist plots of r‐CeCuO_x_ and r‐CuO_x_ show small circles at the high frequency interval, suggesting a solid electrolyte interface membrane (SEI).^[^ ^20^ ^]^ For the r‐Cu, an electric inductive circuit can be observed at high frequency, indicating the low ion capacity on surface. Typically, the r‐CeCuO_x_ shows smaller semicircle on the Nyquist plots measured at −0.93 V than r‐Cu and r‐CuO_x_, suggesting a quick supply of electrons at the interface (Figure S26, Supporting Information). What's more, the LSV curves of the r‐CeCuO_x_ show much higher current densities than those of r‐CuO_x_ and r‐Cu, and the r‐CeCuO_x_ shows lower Tafel slopes than r‐CuO_x_ and r‐Cu, suggesting the improved electron transfer further (Figure S27a,b, Supporting Information).^[^ ^21^ ^]^

Cyclic voltammetry (CV) measurements in CO_2_‐saturated 0.5 M KHCO_3_ were used to study the change of Cu species. We can see that Cu and CuO_x_ show Cu^0^/Cu^+^ and Cu^+^/Cu^2+^ oxidation peaks, which can be attributed to the oxidation of in‐situ formed metallic Cu phase and Cu^+^ oxide, respectively.^[^ ^22^ ^]^ However, the r‐CeCuO_x_ only shows the oxidation peak of Cu^+^/Cu^2+^, suggesting the Cu^+^ is difficult to be reduced in e‐CO_2_RR (Figure S28, Supporting Information).^[^ ^23^ ^]^ CV measurements were held in 1.0 m KOH too and the OH^−^ was used to probe the in‐situ dynamic reconstructed crystal facet (Figure 5b). For r‐CeCuO_x_ and r‐CuO_x_, we can see that the oxidation peaks locate at ≈0.31, ≈0.36, and≈0.56 V, corresponding to the OH^−^ adsorption and Cu oxidation (Cu + OH^−^ → Cu(OH)_ad_ + e^−^) on Cu (100), Cu (110) and Cu (111), respectively,^[^ ^17^, ^24^ ^]^ indicating co‐existence of Cu (100), Cu (110) and Cu (111). For the r‐Cu, only an oxidation peak at≈0.48 V can be seen, indicating only Cu (111) present on r‐Cu. Notably, the OH^−^ adsorption peak of Cu (111) of r‐CeCuO_x_ and r‐CuO_x_ show≈20 mV positive shift compared with that of r‐Cu, suggesting the Cu (111) derived from r‐CeCuO_x_ and r‐CuO_x_ are more stable. Meanwhile, the OH^−^ adsorption peak on Cu (110) of r‐CeCuO_x_ shows ∼15 mV negative shift than that of r‐CuO_x_, indicating the Cu atoms of Cu (110) in r‐CeCuO_x_ have higher activity (Figure 5b). Importantly, r‐CeCuO_x_ shows higher Cu (100)/Cu (110) peak area ratio of than that of r‐CuO_x_, suggesting the CO_2_‐to‐ethylene is more favorable (Figure 5c).^[^ ^25^ ^]^ On the contrary, r‐Cu shows Cu (111) only, that should be favorable for CO_2_‐to‐CH_4_ conversion (Figure 5b,c).^[^ ^17^, ^26^ ^]^

Water cleavage (H_2_O + e^−^→ H + OH^−^) provides proton, which is crucial for CO_2_‐to‐ethylene conversion. The hydrophilic measurement was held on r‐CeCuO_x_, r‐CuO_x_, and r‐Cu after the 70‐min e‐CO_2_RR test to study the surface water affinity (Figure 5d). The contact angle of r‐CeCuO_x_ is≈0^°^, smaller than that of r‐CuO_x_ and r‐Cu, demonstrating its excellent surface hydrophilicity,^[^ ^27^ ^]^ which could assist the exposure of active sites to the electrolyte and reduce the electrode‐electrolyte contact resistance at the interface (Figure 5d). The density of local proton donors near the surface of catalyst can be estimated by the HCO_3_^−^/CO_3_^2−^ peak height ratio.^[^ ^28^ ^]^ The r‐CeCuO_x_ and r‐Cu show smaller HCO_3_^−^/CO_3_^2−^ peak height ratio than r‐CuO_x_, indicating more proton donors are available (Figure 5e; Figure S30a–c, Supporting Information).^[^ ^29^ ^]^ The high proton concentration leads to a decrease in the local pH at the interface that favors the water cleavage, which can be further confirmed by the reduced Tafel slopes measured without CO_2_ (Figure S27c, Supporting Information).^[^ ^30^ ^]^ Additionally, the acidic condition enables CO_2_ on the surface and avoids its conversion to carbonate.^[^ ^31^ ^]^ ESR measurement was conducted to further investigate the characteristics of hydrogen radical species (H^*^) using 5, 5‐dimethyl‐1‐pyrroline‐N‐oxide (DMPO) as probe reagent.^[^ ^32^ ^]^ Only the characteristic peaks of DMPO‐CO_3_^*^ radicals (DMPO‐*C) can be seen in KHCO_3_ solution (Figure S29, Supporting Information). For the electrolytes after 70‐min electrolysis at −0.93 V, nine characteristic peaks of DMPO‐*H are visible, confirming the formation of H* (Figure S29, Supporting Information).^[^ ^32c^ ^]^ Quasi in‐situ ESR experiment was conducted after 70‐min test at −0.93 V to study the H* on electrocatalyst further. We can see that the characteristic peaks of DMPO‐*C and DMPO‐H species adduct (DMPO‐*H) exist in the spectra of r‐CeCuO_x_, r‐CuO_x_ and r‐Cu, which come from KHCO_3_ in electrolyte and H species on the electrode, respectively (Figure 5f). Notably, the DMPO‐*H peaks for r‐CeCuO_x_ show higher intensities than those for r‐CuO_x_ and r‐Cu, indicating more *H on r‐CeCuO_x_ surface. Importantly, magnetic fields of DMPO‐*H on r‐CeCuO_x_ are positively shifted compared with those of r‐CuO_x_ and r‐Cu, underscoring the H* on r‐CeCuO_x_ is more active,^[^ ^33^ ^]^ which should be responsible for the excellent e‐CO_2_RR performance (Figure 5f). Besides the high concentration of active H*, the adsorbed OH^−^ reduces the rate of Heyrovsky step (H* + H_2_O + e^−^→ H_2_ + OH^−^). Herein, in‐situ Raman spectra measured at 30^th^ second after the bias is terminated show that the OH^−^ adsorption peak of r‐CeCuO_x_ is higher than those of r‐CuO_x_ and r‐Cu, suggesting the adsorbed OH^−^ on r‐CeCuO_x_ is higher (Figure 5g).^[^ ^13^, ^34^ ^]^ Therefore, the hydrogen gas production in the Heyrovsky step on r‐CeCuO_x_ may be suppressed.

Carbon reactions, including C_1_ intermediate formation, hydrogenation, and C‐C coupling/C═C formation, are keys of carbon skeleton construction. In‐situ Raman scatting spectroscopy shows bridge and atop sites of CO adsorption at 1800–2000 and 2000–2200 cm^−1^ (named *CO_bridge_ and *CO_atop_), respectively, for all the samples (Figure S30c–e, Supporting Information).^[^ ^35^ ^]^ The r‐CuO_x_ shows lowest *CO_bridge_ and *CO_atop_ among the three samples, indicating fewer CO intermediate, where the formation of carbon skeleton of ethylene may be hampered (Figure 5h). Further, we resorted in‐situ attenuated total reflection Fourier‐transform infrared spectroscopy (FT‐IR ATR) measurements on r‐CeCuO_x_, r‐CuO_x_ and r‐Cu at different potentials. We can see that characteristic peaks of C═C, bending of CO_3_^2−^ (δCO_3_^2−^), COCHO, COOH, stretching vibration of CO_3_^2−^ (vCO_3_^2−^) and O‐H of water can be observed in the spectra of r‐CeCuO_x_ and r‐CuO_x_ measured from −0.63 to −1.13 V (Figure 5i–j).^[^ ^13^, ^26^, ^35^, ^36^ ^]^ Notably, the intensities of C═C, COCHO and COOH characteristic peaks gradually increases when the potential becomes negative, acknowledging more crucial intermediates for C═C formation, C‐C coupling, and C_1_ intermediates (typically CO) generation, respectively (Figure 5i–k).^[^ ^36^, ^37^ ^]^ It is worth noticing that the COCHO is almost negligible, and C═C characteristic peaks of r‐CuO_x_ are weaker than those on r‐CeCuO_x_ obviously, coincident with the low FE ethylene. For r‐Cu, characteristic peaks of δCO_3_^2−^, vCO_3_^2−^ and O‐H of water, COOH and CHO can be seen. The intensity of COOH peak decreases with potential become negative, suggesting its fast consumption (Figure 5f). Notably, negligible COCHO and C═C peaks can be seen throughout the entire potential interval from −0.63 to −1.13 V, indicating the C‐C coupling and C═C formation reactions are sluggish (Figure 5h). The in‐situ FT‐IR ATR spectra of r‐CeCuO_x_, r‐CuO_x_ and r‐Cu measured at −0.93 V show that: (i) The COOH and CHO characteristic peaks of r‐CeCuO_x_ is stronger than those of r‐CuO_x_, suggesting the COOH → CHO conversion is enhanced. Meanwhile, the COOH and CHO of r‐CeCuO_x_ show red shift compared with that of r‐Cu, suggesting they are highly activated (Figure 5l). (ii) A stronger COCHO characteristic peak can be observed in spectra of r‐CeCuO_x_ than those of r‐CuO_x_ and r‐Cu, suggesting that C_2_ intermediates generation may benefit. Additionally, the C═C characteristic peak of r‐CeCuO_x_ is blue‐shift compared with that of r‐CuO_x_, indicating the ethylene generation shall be more beneficial (Figure 5l). Therefore, r‐CeCuO_x_ has the best performance on the CO_2_‐to‐C_2_H_4_ conversion.

Based on all the experiment results and discussions above, we can see that the Cu^0^/(Cu^+^+Cu^2+^) ratio of r‐CeCuO_x_ (0.856) is closer to 1 than those of r‐Cu (1.288) and r‐CuO_x_ (0.395), endowing optimized electron distributions on Cu^0^ and Cu^+^, which enhance the ethylene formation. The dominant Cu^0^ ions with rich electrons in r‐Cu shall benefit the C_1_ intermediate generation, COOH consumption and CHO stabilization, but the rare Cu^+^ and Cu^2+^ ions make the C_2_ intermediate conversion, especially C‐C coupling, becoming sluggish, leading to the CH_4_ production. The abundant Cu^+^ and Cu^2+^ ions and the rare Cu^0^ in r‐CuO_x_ make the generations of C_1_/C_2_ intermediates sluggish, resulting in the H_2_ production (Figure 5m).

## 3 Conclusion

In this work, we fabricate Ce‐incorporated Cu oxide as pre‐catalyst (CeCuO_x_) by simple treatments of Cu. The CeCuO_x_ shows excellent ethylene FE of 55.39% with a j_ethlyene_ of −39.50 mA cm^−2^ as well as high stability up to 590 h at −0.93 V. We find that the incorporated Ce is leached, and the metallic oxygen‐contained Cu with optimized electronic structure is obtained, promoting the CO_2_‐to‐ethylene conversion, which can be attributed by: i) enhanced water cleavage and abundant active hydrogen species for the hydrogenation and deoxygenation of CO_2_ and intermediates, ii) OH^−^‐enriched micro environment for the prevention of competitive HER, and iii) improved specific C_1∼2_ intermediates generation and activation for the ethylene production. Our findings may provide insightful understanding on the relationship between leaching‐induced reconstructed structure and catalytic performance improvement and guide the design of novel electrocatalyst for effective e‐CO_2_RR as well as other electrocatalysis.

## Conflict of Interest

The authors declare no conflict of interest.

## Supporting information



Supporting Information

## Data Availability

The data that support the findings of this study are available from the corresponding author upon reasonable request.
