# Supplementary material for: Reconstructed Copper Compound by Cerium Leaching for Enhanced Electrochemical CO2‐to‐Ethylene Conversion
Source: Adv Sci (Weinh). 2026 Jan 2;13(6):e04094. doi: 10.1002/advs.202504094 (PMC12866719; doi:10.1002/advs.202504094)
Supplement: Supplementary file 1 — Supporting Information [file ADVS-13-e04094-s001.docx]

**Supporting Information**

**Reconstructed Copper Compound by Cerium Leaching for Enhanced Electrochemical CO_2_-to-Ethylene Conversion**

Jinxian Feng ^a^, Chunfa Liu ^a^, Yu-Xuan Xiao ^a^, Lun Li ^a^, Keyu An ^a^, Sen Ding ^a^, Weng Fai Ip ^b^, Daniel H. C. Chua ^c^, and Hui Pan^* a, b^

*^a^ Institute of Applied Physics and Materials Engineering, University of Macau, Macao S. A. R., China*

*^b^ Department of Physics and Chemistry, Faculty of Science and Technology, University of Macau, Macao S. A. R., China*

*^c^ Department of Materials Science and Engineering, National University of Singapore, Singapore*

*Corresponding author: Hui Pan, [huipan@um.edu.mo](mailto:huipan@um.edu.mo) (email), +853-88224427 (tel.), +853-88222454 (fax).

**Experimental method**

**Chemicals and reagents.** All chemicals were utilized as received without any treatment. Deionized (DI) water was supplied by a Barnstead Nanopure water system (resistivity: 18.3 MΩ/cm) and was used for the preparation of all aqueous solutions. Cesium nitrate (Ce(NO_3_)_3_·6H_2_O, >99%), lanthanum nitrate (La(NO_3_)_3_·3H_2_O, >99%), aluminum nitrate (Al(NO_3_)_3_·6H_2_O, >99%), sodium hydroxide (NaOH, >97%), hydrochloric acid (HCl, 36%), potassium bicarbonate (KHCO_3_, >99%) and ethanol (C_2_H_5_OH, >99%) were purchased from Aladdin, Co. Ltd. Copper plate (99.9% purity, thickness = 0.1 mm) was purchased from Non-ferric metal Co. Ltd., Xi’an, Shanxi.

**Electrocatalyst fabrication.** The Ce incorporated Cu oxide was prepared via electrochemical oxidation. Typically, 15 mL 0.1 M KHCO_3_ was used as electrolyte, and a piece of Cu plate (4 cm × 2 cm) after surface cleaning (firstly polished by abrasive paper, then soaked in 1 M NaOH solution for 20 min to remove oil on surface, and finally risen in 1 M HCl with ultrasonic to remove residual NaOH and oxide on surface) was used as anode, a piece of graphite plate was used as cathode, with the current density is 2.5 mA/cm^2^, and the electrochemical anodization time is 90 minutes. After that, the anodized Cu plate was then soaked in different concentrations of Ce(NO_3_)_3_ (2, 5, 10 and 20 mM) and different inorganic salts (La(NO_3_)_3_ and Al(NO_3_)_3_) of 10 mM with vigorous stirring for 12 hours, and annealing in air for 2 hours at 300°C by using Muffle-oven (Ke Jing Co. Ltd. Hefei, OTF-1200X).

**Materials characterizations.** The crystal structures were characterized on a powder X-ray diffraction (XRD) (Rigaku rotating anode diffractometer with a mono chromated Cu Kα X-ray source). The morphology and chemical composition were determined by scanning electron microscopy (SEM) (ZEISS-Merlin) and transmission electron microscope (TEM) (JEM-F200) coupled with energy dispersive spectrometer (EDS). Raman spectra were recorded by a confocal laser Raman system using 532 nm laser as excitation wavelength, with an acquisition time of 10 s. X-ray photoelectron spectroscopy (XPS) spectra were collected on a Thermo Fisher Scientific Theta Probe with Mg Ka (hν = 1253.6 eV) as the excitation source.

**Electrochemical test.** 0.5 M KHCO_3_ saturated by CO_2_ was used as electrolyte. The electrochemical tests towards CO_2_ reduction performance were conducted on an electrochemical workstation (CHI 660D). The as-prepared samples were directly used as the working electrodes, a Ti mesh with Ru-Ir oxide coating was utilized as the counter electrode, and a Hg/Hg_2_Cl_2_ in saturated KCl was used as the reference electrode. Prior to the CO_2_ reduction, the electrolyte was plugged by CO_2_ gas for at least 15 minutes to fully saturated and remove the residual air. The galvanostatic tests at different potentials were held for 3 h. All the potentials were converted to the reversible hydrogen electrode (RHE) via the Nernst equation (E vs. RHE = E vs. Hg/Hg_2_Cl_2_ + 0.0591 × pH + 0.241 V). The electronic impedance spectroscopy (EIS) plots were measured in the frequency 0.01-10^6^ Hz with an amplitude of 10 mV.

The turnover frequency (TOF) is calculated according to Equation (1):^[1]^

TOF = n_ethylene_/(n_Cu_ × t) (1)

n_ethylene_ is number of moles of detected ethylene and n_Cu_ is the measured number of moles of Cu present on the cathode through ICP-MS. t represents reaction time, s.

**Products Analysis.** The liquid products were analyzed using high-performance liquid chromatography (HPLC, Agilent 1260). The flow phase is de-ionized water, the solution volume of each injection batch is 15 μL with the flow rate 0.6 mL/minute. The CO and gaseous flammable carbon-based gaseous products were measured by gas chromatography (GC) (7890B, Agilent Technologies) with a flame-ionized detector (FID), and the hydrogen gas was measured by GC with a thermal conductivity detector (TCD).

The FE of the products were calculated as the Equation (2):

FE = $\frac{n}{n_{0}}$ (2)

n represents the generated molar amounts of products, mol; n_0_ represents the molar of products generate in principle, mol. n_0_ can be calculated as Equation (3):

n_0_ = $\frac{Q}{\mathrm{zF}}$ (3)

Q represents the Column number, C; z represents the number of electron transfer. For HCOO^-^, CO and H_2_, n=2; for CH_4_, n=8, for C_2_H_4_, n=12.

The partial current density is calculated as Equation (4):

j = $\frac{Q}{t}\times FE$ (4)

t represents the reaction time, s.

**In-situ analysis.** In-situ Raman spectrum was measured by using Teflon in-situ Raman cell, with 1 mm thickness of 0.5 M KHCO_3_ electrolyte saturated by CO_2_ as electrolyte. The Raman excitation wavelength is 532 nm, with 100% power ratio and the acquisition time of 20 s. The current intensities were directly controlled by an electrochemical workstation (CHI 760E). In-situ attenuated total reflection Fourier-transform infrared (FT-IR ATR) spectrum was measured by using electrolysis cell with three-electrode configuration. The obtained materials were used as the working electrode directly, with a Pt wire as the counter electrode and a saturated Ag/AgCl as the reference electrode. The cell was integrated into a Shimadzu Xross FT-IR spectrometer equipped with a liquid nitrogen-cooled MCT detector and attenuated total reflection device (Linglu Co. Ltd., Shanghai, China) using single crystal silicon as ATR crystal. Before the tests, the working electrode was reduced in CO_2_-saturated 0.5 M KHCO_3_ for 70 minutes at -0.93 V. Each spectrum consisted of 64 single beams with a resolution of 8 cm^−1^. The spectrum was collected at 0 V vs. RHE with CO_2_-saturated 0.5 M KHCO_3_ as a reference.

**Figure S1**. XRD patterns of oxidized Cu and Ce-incorporated oxidized Cu.


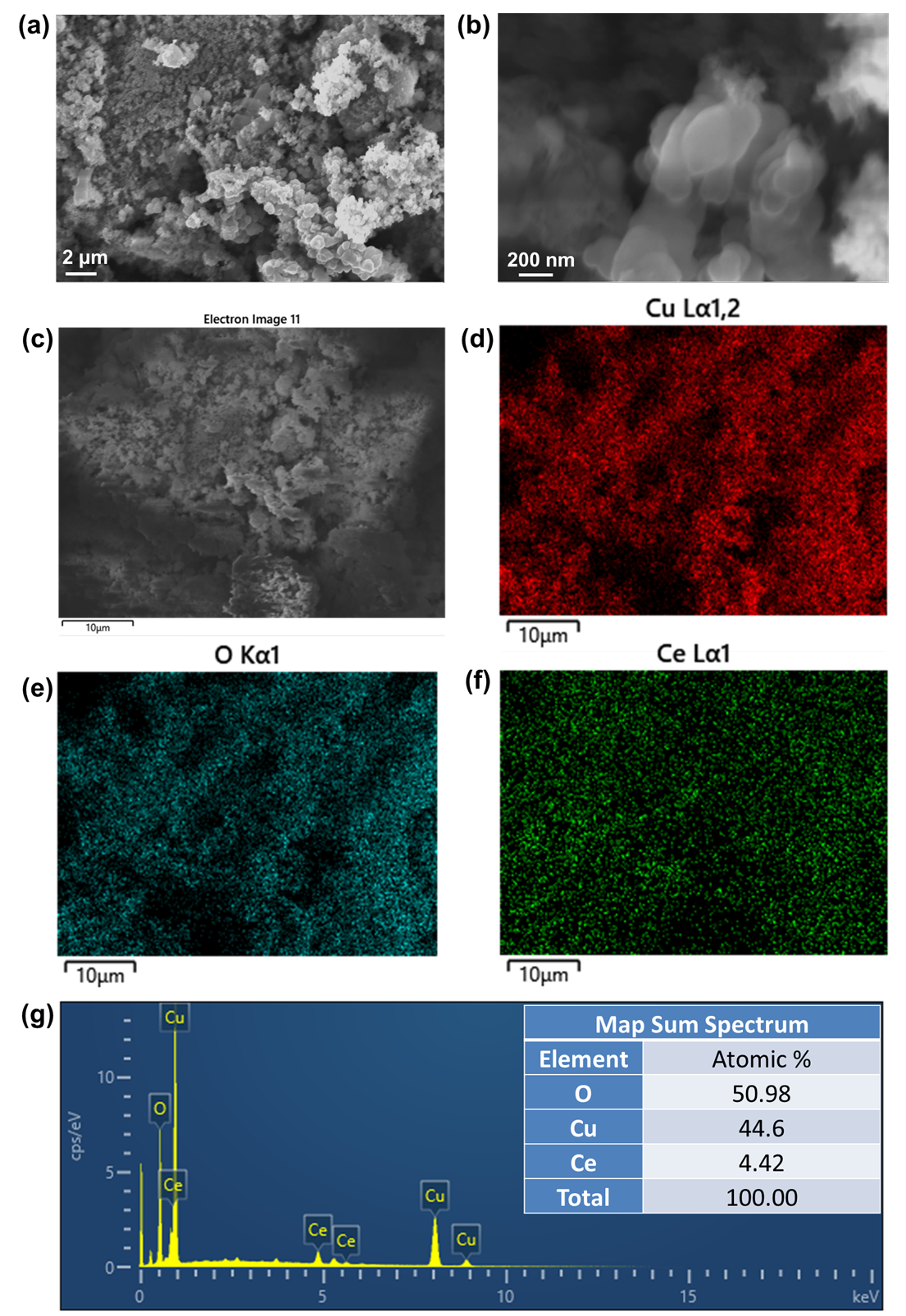


**Figure S2.** (a-b) SEM images of CeCuO_x_ pre-catalyst. Element distribution mapping: (c) SEM image of selected area, (d) O, (e) Cu and (f) Ce. (g) EDS spectrum with element content table.


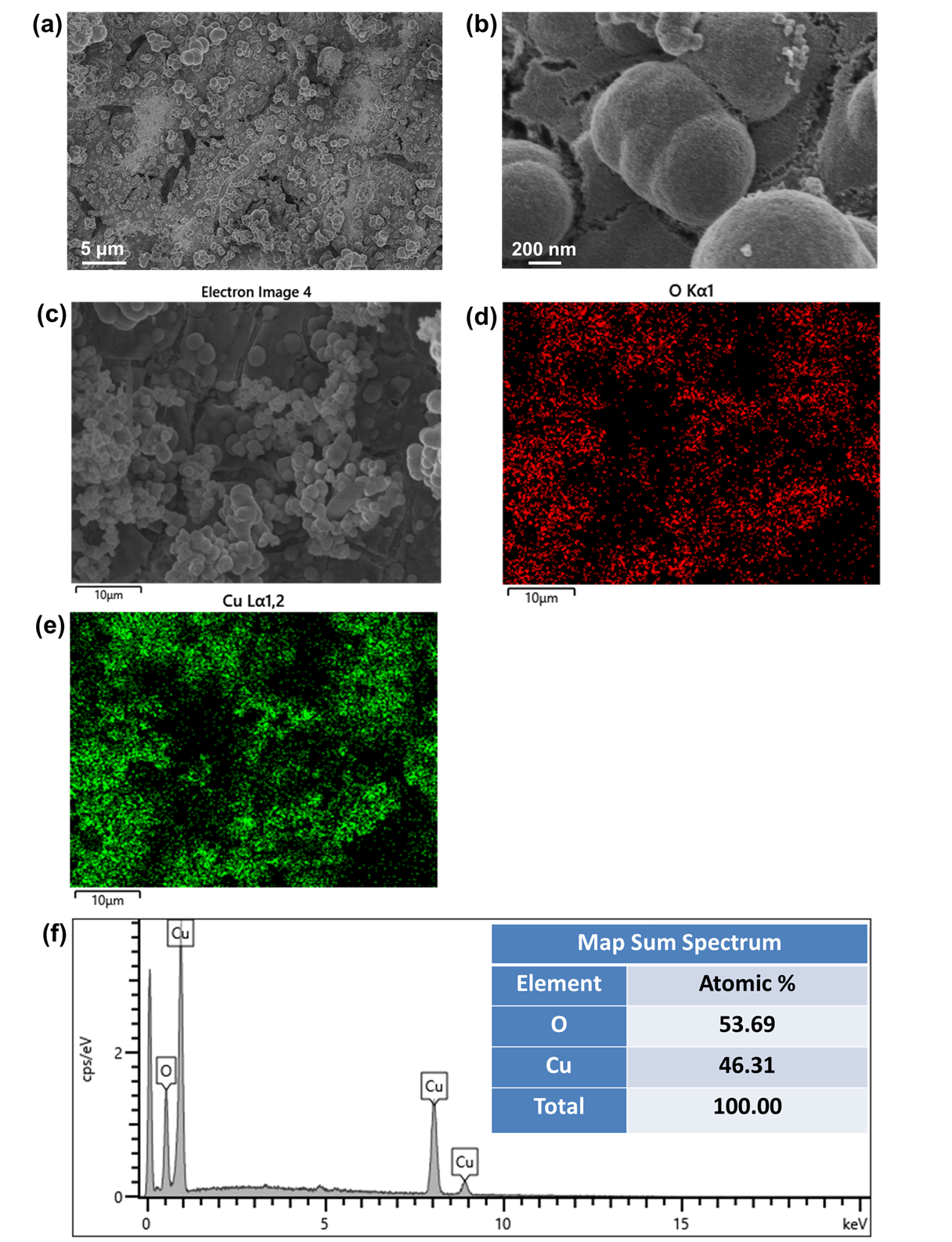


**Figure S3.** (a-b) SEM images of CuO_x_ pre-catalyst. Element distribution mapping: (c) SEM image of selected area, (d) O, and (e) Cu. (f) EDS spectrum with element content table.


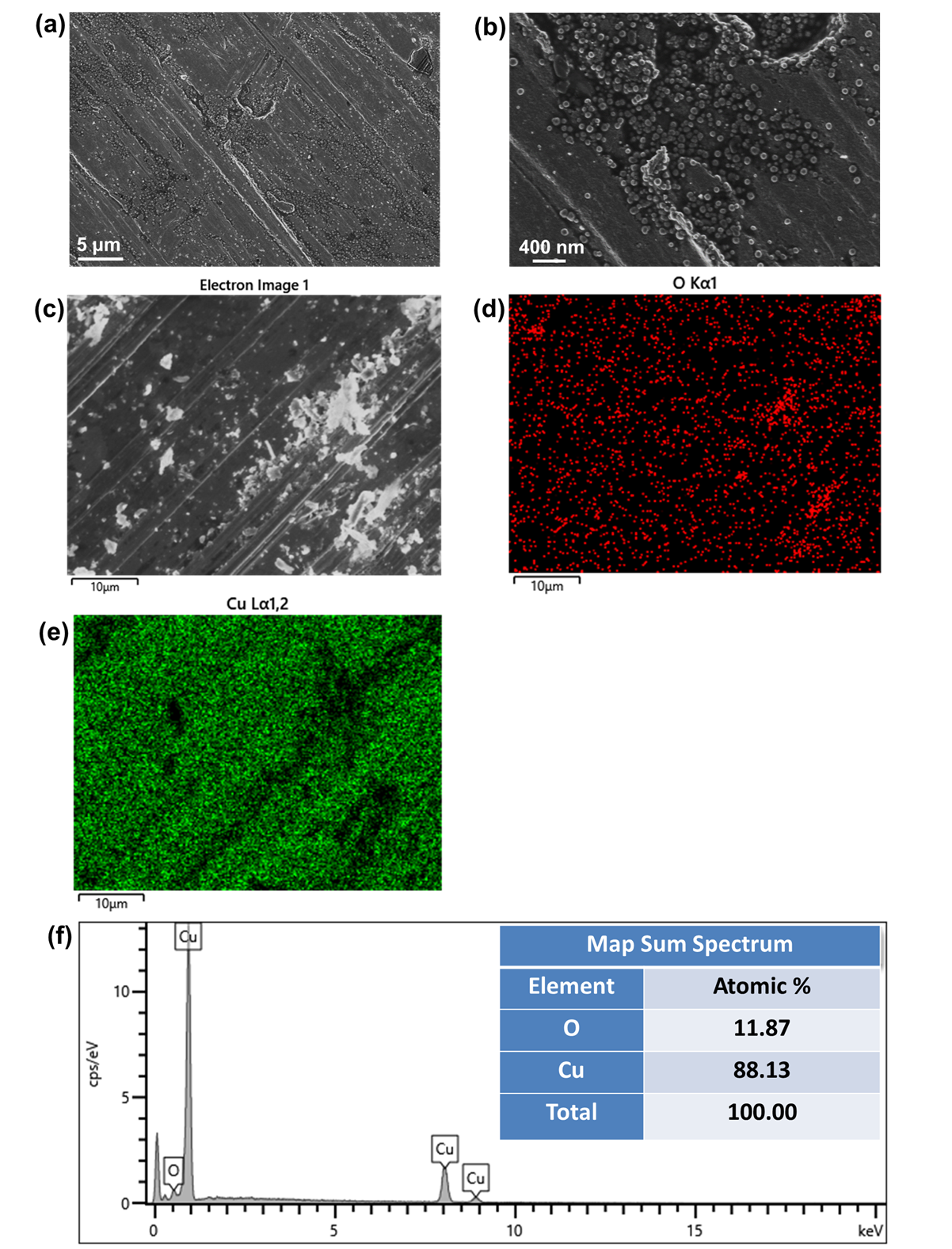


**Figure S4.** (a-b) SEM images of Cu pre-catalyst. Element distribution mapping: (c) SEM image of selected area, (d) O, (e) Cu and (f) EDS spectrum with element content table.


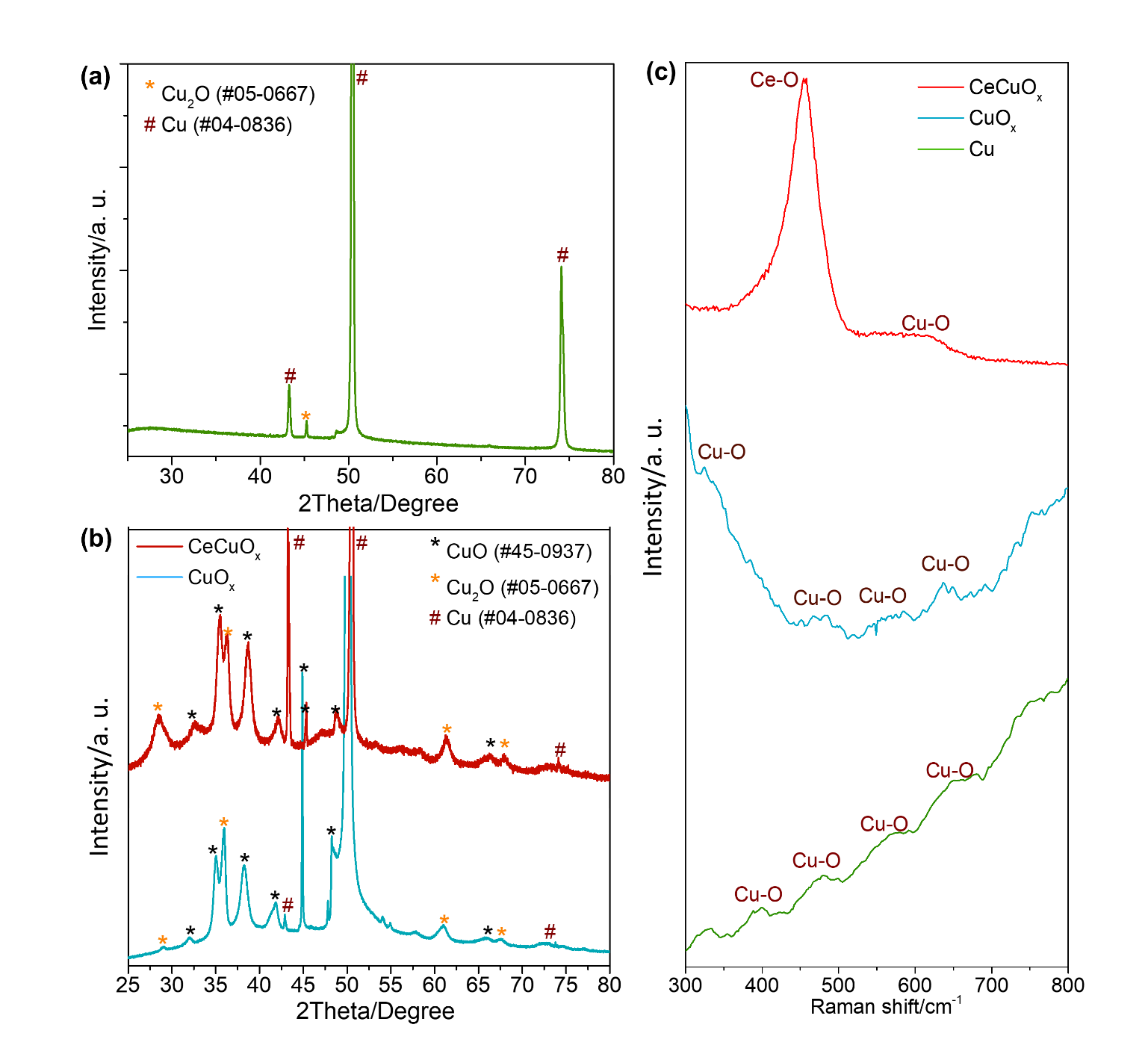


**Figure S5.** XRD patterns of pre-catalysts: (a) Cu and (b) comparison of CeCuO_x_ and CuO_x_. (c) Raman spectrum of CeCuO_x_, CuO_x_ and Cu pre-catalysts.


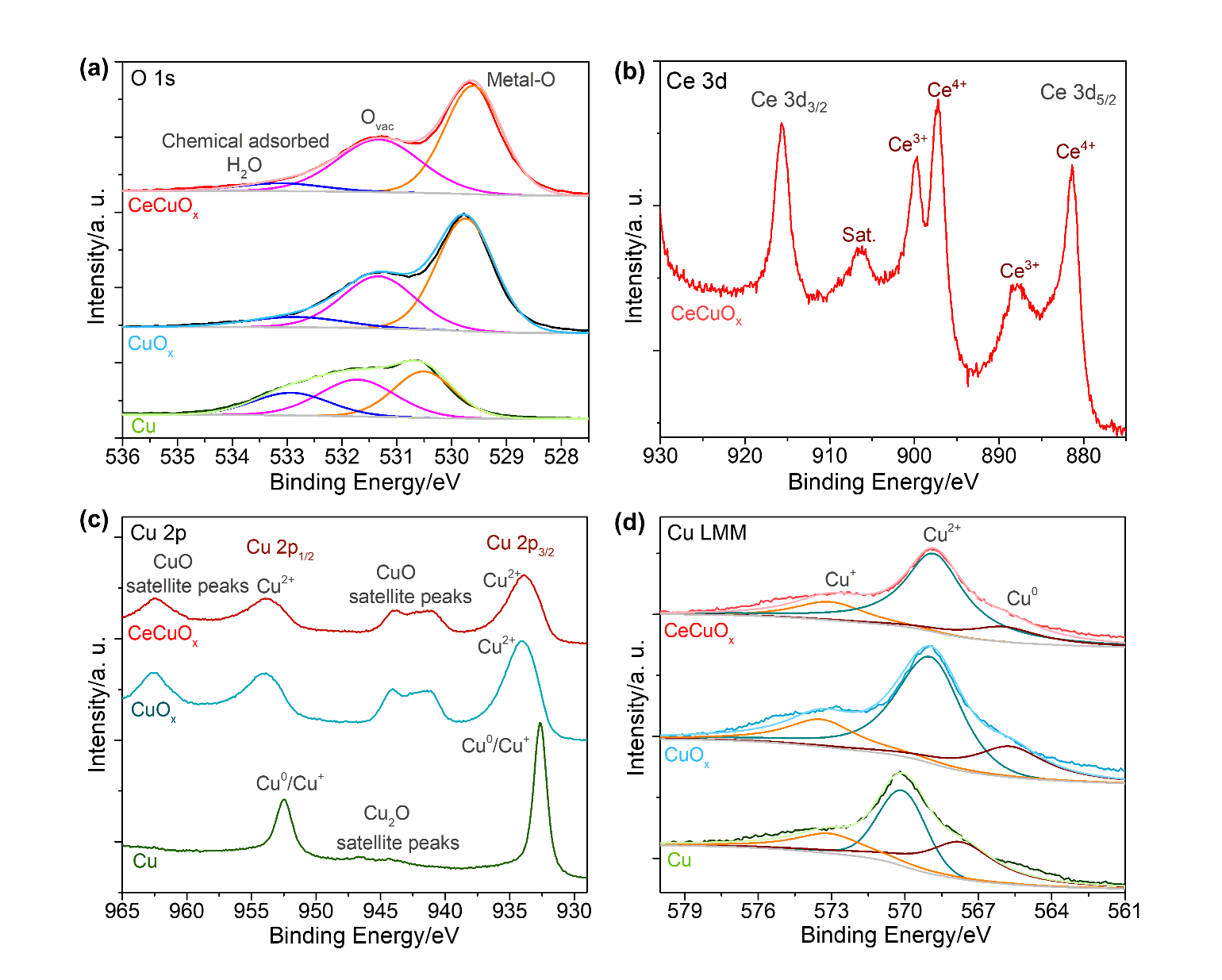


**Figure S6.** XPS spectra of pre-catalysts: (a) O 1s of CeCuO_x_, CuO_x_ and Cu, (b) Ce of CeCuO_x_, (c) Cu 2p, and (d) Cu LMM.


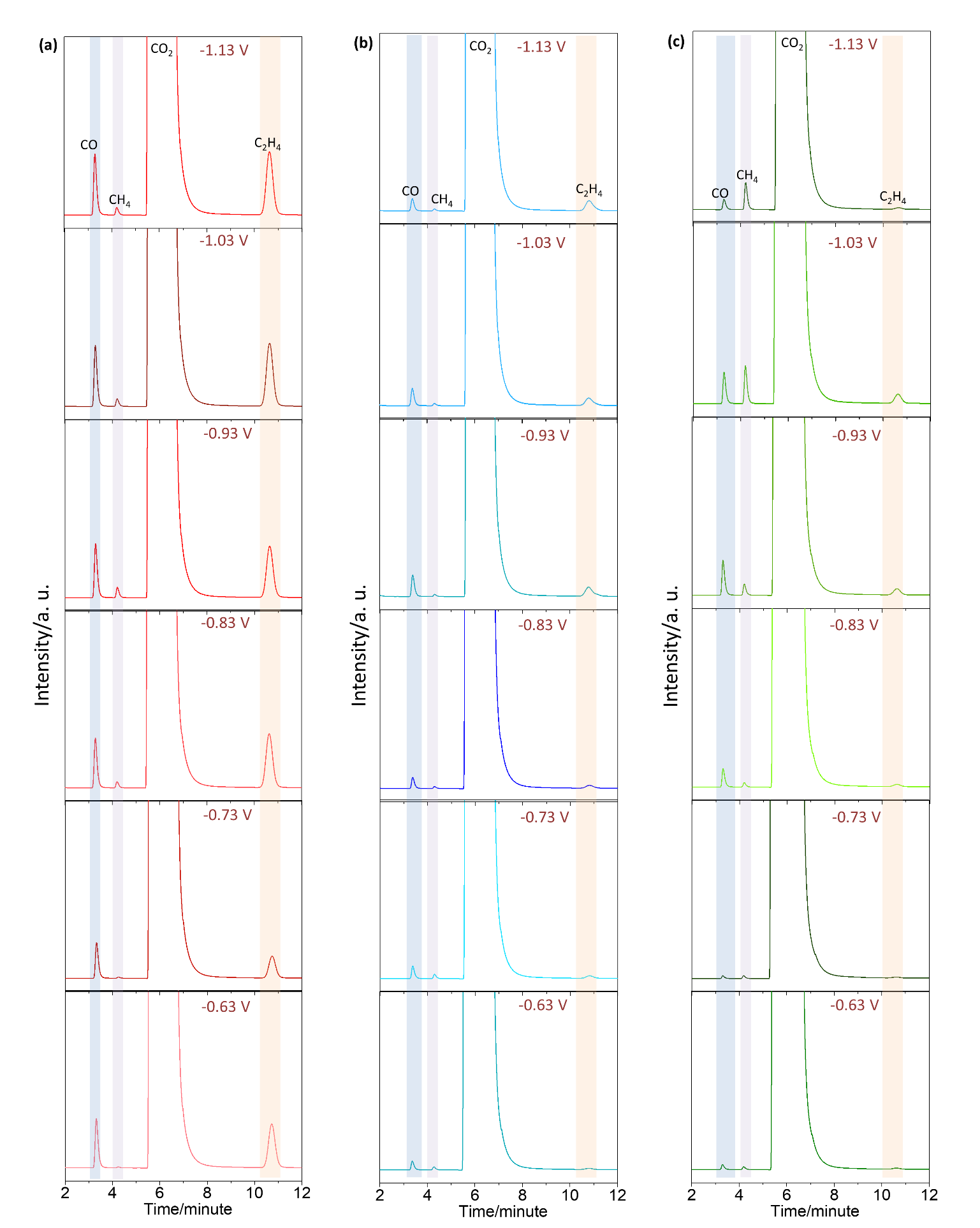


**Figure S7.** GC chromatography of gas products at various potentials: (a) CeCuO_x_, (b) CuO_x_ and (c) Cu.


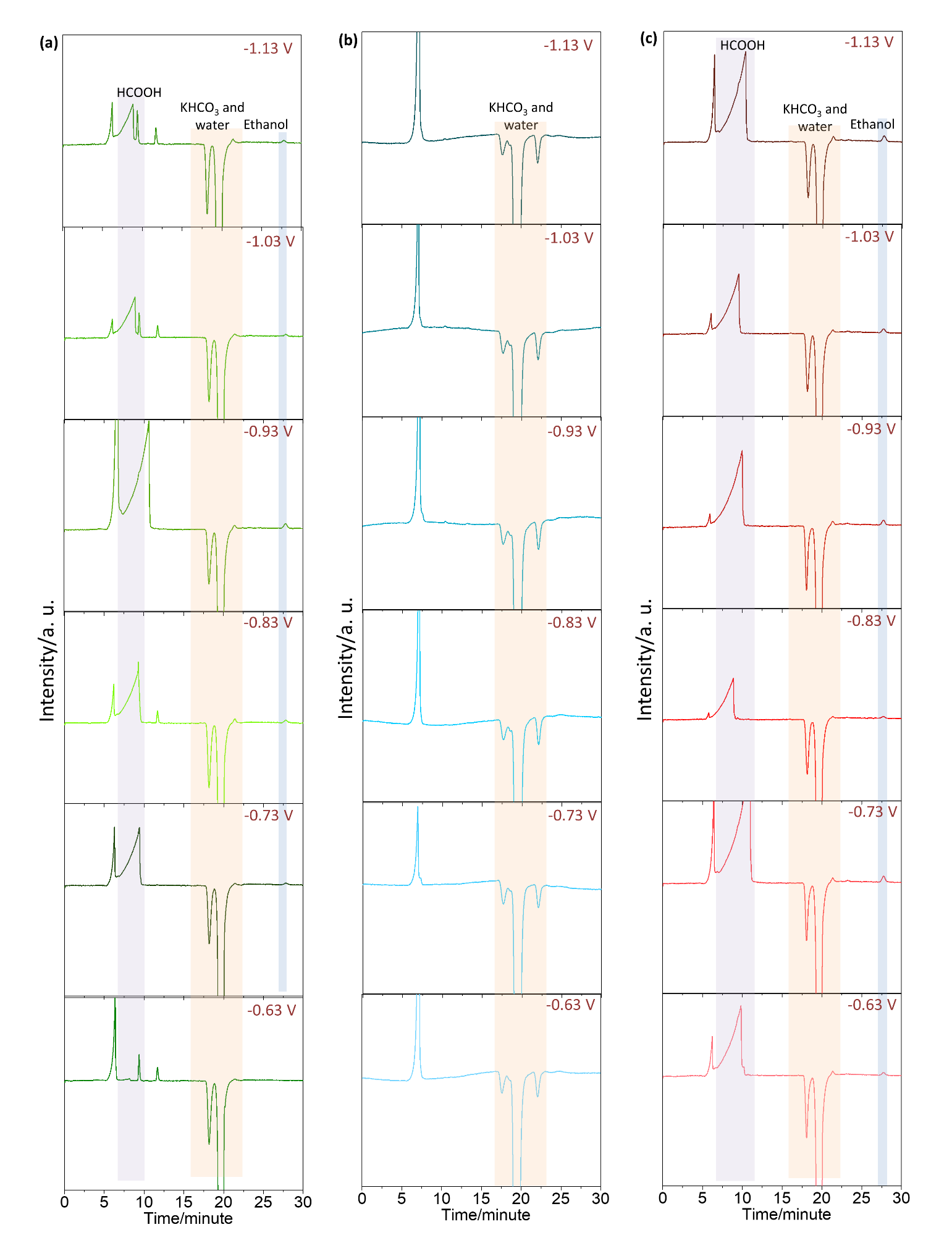


**Figure S8.** HPLC chromatography of liquid products at various potentials: (a) CeCuO_x_, (b) CuO_x_ and (c) Cu.

**Table S1.** Cu contents and n_Cu_ of r-CeCuO_x_, r-CuO_x_ and r-Cu.

| Sample | r-CeCuO_x_ | r-CuO_x_ | r-Cu |
| --- | --- | --- | --- |
| Cu content/μg cm^-2^ _catalyst_ | 13.34 | 17.3 | 34.4 |
| n_Cu_/mol (per cm^2^) | 2.10 × 10^-7^ | 2.72 × 10^-7^ | 5.41 × 10^-7^ |

**Figure S9.** TOF values of CeCuO_x_, CuO_x_ and Cu at different potential.


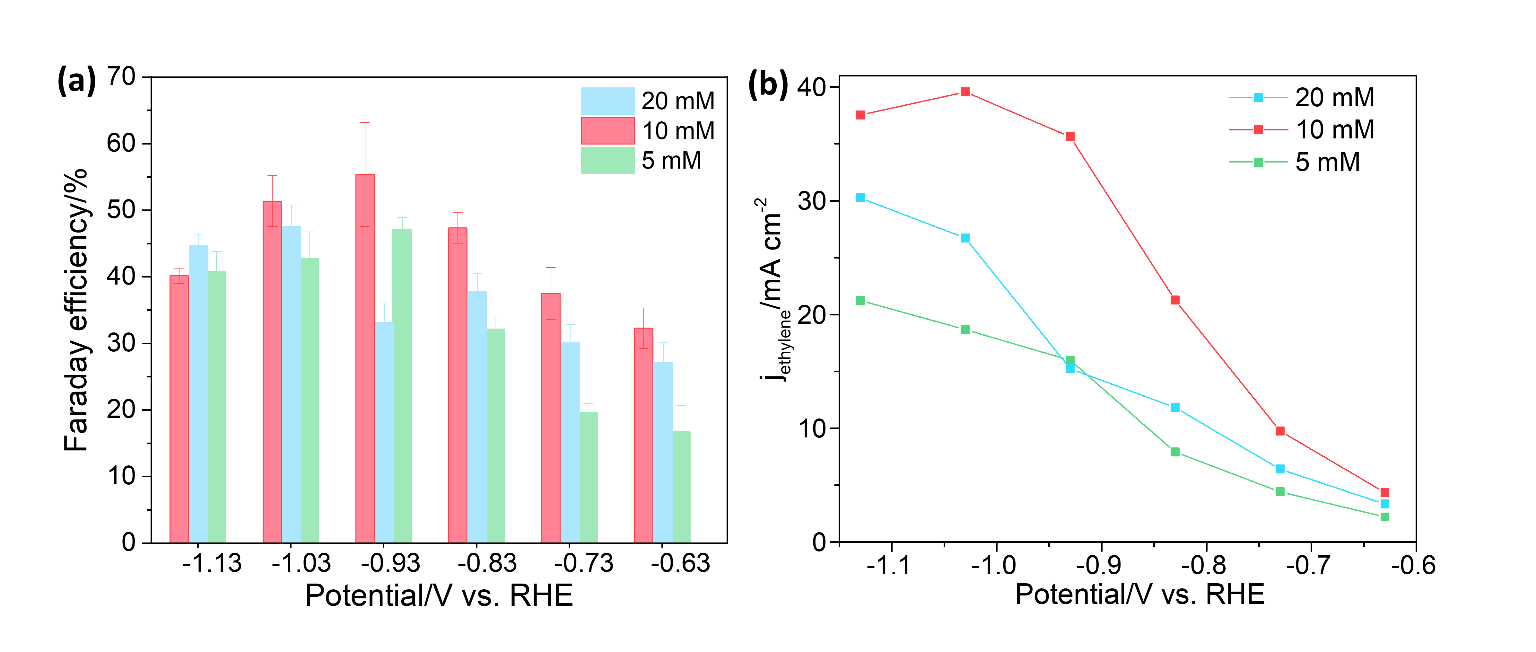


**Figure S10.** (a) FE ethylene and (b) j_ethylene_ of CeCuO_x_ using different Ce(NO_3_)_3_ concentrations.


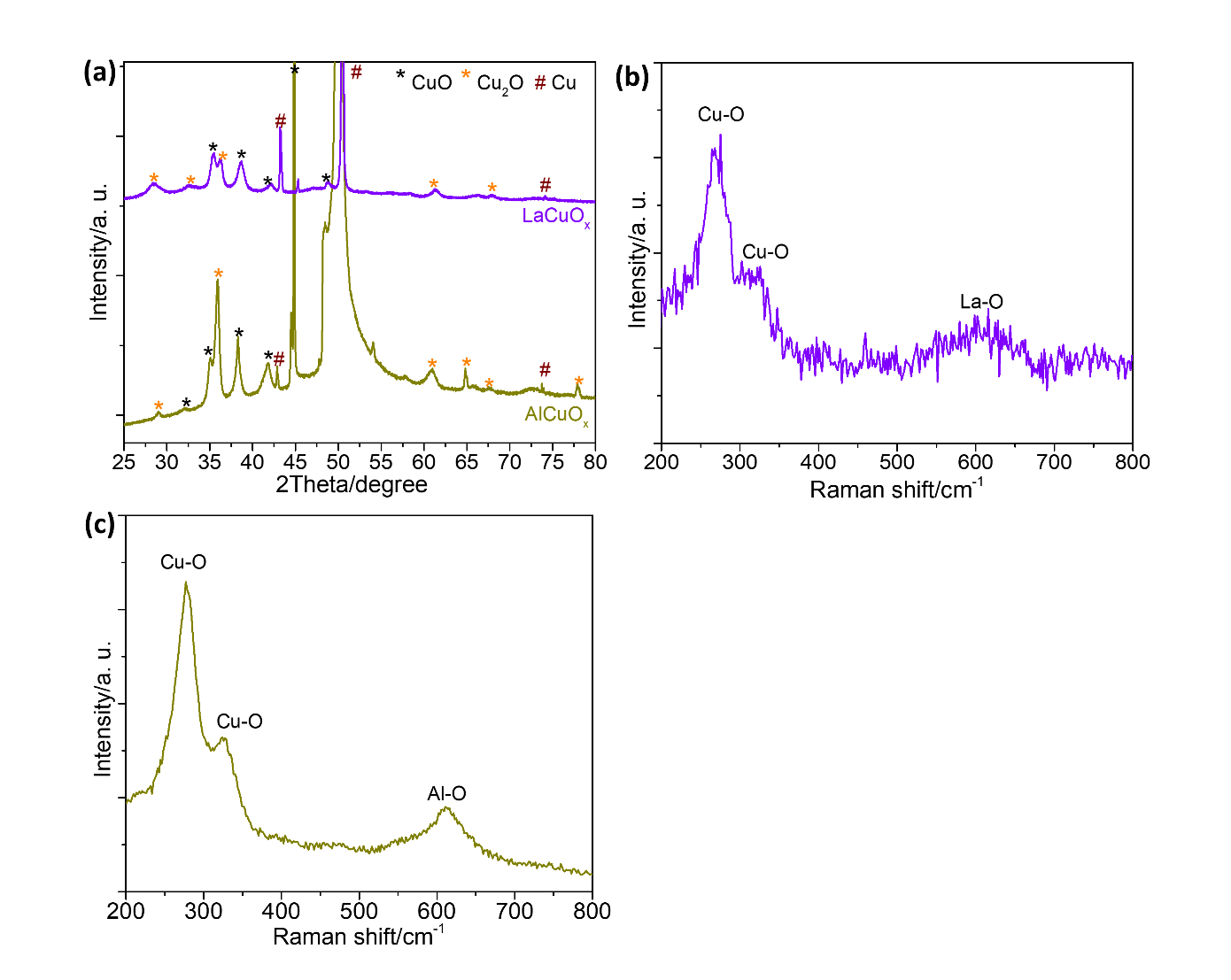


**Figure S11.** (a) XRD patterns of AlCuO_x_ and LaCuO_x_ pre-catalysts. Raman spectrum of (b) LaCuO_x_ and (c) AlCuO_x_ pre-catalysts.

**
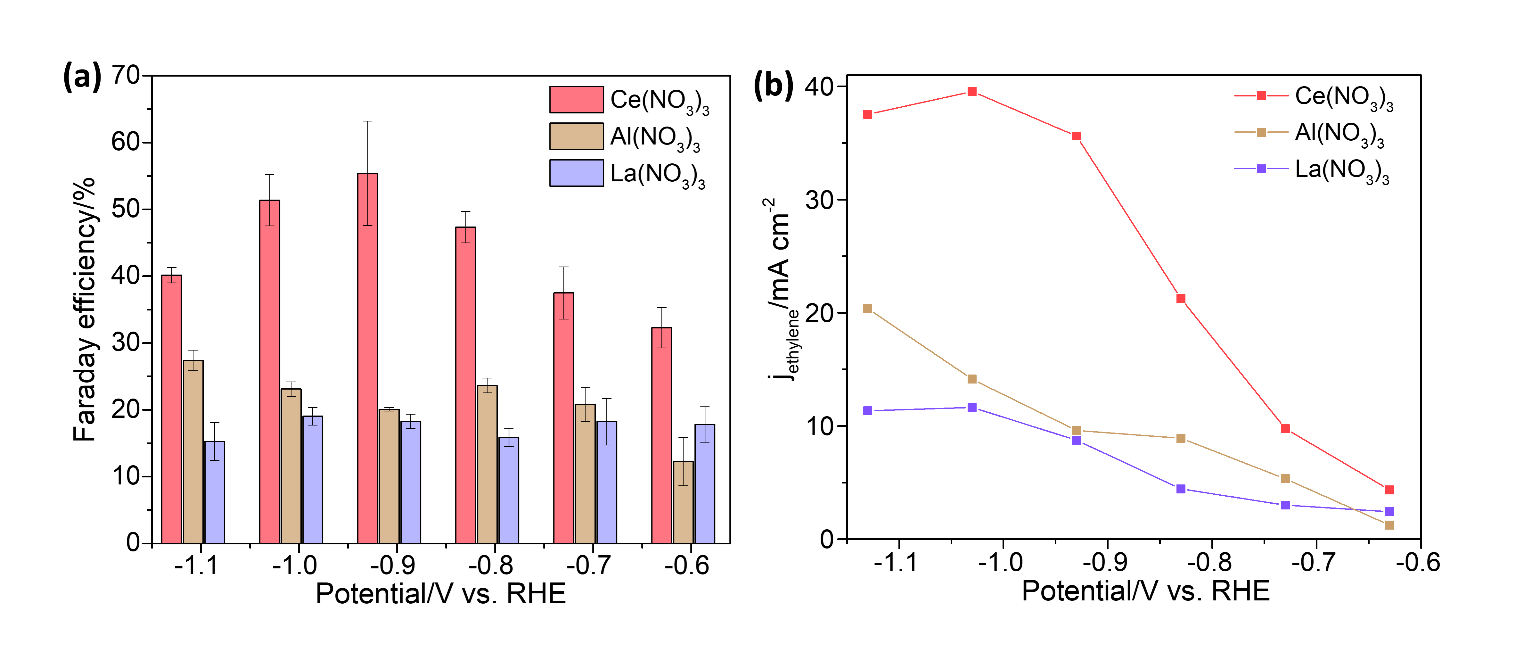
**

**Figure S12.** (a) FE ethylene and (b) j_ethylene_ using 10 mM Ce(NO_3_)_3_, Al(NO_3_)_3_ and La(NO_3_)_2._

**Table S2.** Comparison of CeCuO_x_ CO_2_-to-ethylene performance to other electrocatalysts with similar composition.

| **Electrocatalyst** | **Electrolyte (cell)** | **FE ethylene**  **(Potential/V vs. RHE)** | **j_ethylene_/ mA cm^-2^ (Potential/V vs. RHE)** | **Reference** |
| --- | --- | --- | --- | --- |
| CeCuO_x_ | 0.5 M KHCO_3_ | 37.48 (-0.73)  47.34 (-0.83)  55.39 (-0.93)  51.37 (-1.03)  40.15 (-1.13) | -9.76 (-0.73)  -21.34 (-0.83)  -35.62 (-0.93)  -39.50 (-1.03)  -37.53 (-1.13) | Our work |
| Ce/CuO_x_ | 1 M KOH | 32 (-1.0)  27 (-1.1)  40 (-1.2) | -43 (-1.0)  -42 (-1.1)  -71 (-1.2) | *Angew. Chem. Int. Ed.* 2024, e202419796 |
| Cu-MOF | 0.5 M KHCO_3_ | 27.5 (-0.9)  37.5 (-1.0)  40 (-1.05)  49.6 (-1.15) | -5.2 (-0.9)  -7 (-1.0)  -9.2 (-1.05)  -13.5 (-1.15) | *Chem. Eng. J.* (2024), 499, 156694. |
| CeO_2_/CuO | 0.1 M KCl | 4 (-0.95)  9 (-1.05)  34 (-1.15) | -0.08 (-0.95)  -0.27 (-1.05)  -1.53 (-1.15) | *Carbon Energy.* 2024, e588. |
| CuNNAs | 0.1 M KHCO_3_ | 19 (-1.1)  27 (-1.2) | -1.78 (-1.1)  -18.23 (-1.2) | *Nano Lett.* 2022, 22, 5, 1963. |
| CuOHFCl | 0.5 M KHCO_3_ | 21 (-0.9)  36.3 (-1.0)  33 (-1.05)  25 (-1.1) | -2.31 (-0.9)  -6.53 (-1.0)  -10.56 (-1.05)  -9.38 (-1.1) | *Angew. Chem. Int. Ed.* 2022, 61, e202111700. |
| Wrinkled Cu | 0.1 M KHCO_3_ | 3 (-0.9)  14 (-1.0)  16 (-1.1) | -0.06 (-0.9)  -0.518 (-1.0)  -1.6 (-1.1) | *ACS Catal.* 2021, 11, 9, 5658. |
| Ce-Cu_2_O | 0.5 M KHCO_3_ | 8.5 (-1.0)  12 (-1.1)  15.5 (-1.2)  25 (-1.3) | -1.02 (-1.0)  -2.16 (-1.1)  -3.57 (-1.2)  -7.5 (-1.3) | *ACS Nano* 2023, 17, 13974. |
| CuBtZ | 0.5 M KHCO_3_ | 5 (-1.1)  23 (-1.2)  42 (-1.3) | -0.22 (-1.5)  -1.334 (-1.6)  -2.73 (-1.7) | *J. Am. Chem. Soc.* 2022, 144, 29, 13319 |
| S-HKUST-1 | 0.1 M KHCO_3_ | 38 (-1.12) | -2.14 (-1.12) | *Angew. Chem. Int. Ed.* 2022, 61, e202111700 |
| PROD-Cu-I | 0.1 M KHCO_3_ | 13 (-1.05)  17 (-1.13)  18 (-1.2) | -1.04 (-1.05)  -2.21 (-1.13)  -2.88 (-1.2) | *Small* 2024, 2401530 |
| CuO–CeO_2_/CB | 0.1 M KHCO_3_ | 15 (-0.9)  40 (-1.0)  50 (-1.1) | -0.75 (-0.9)  -1.8 (-1.0)  -3.7 (-1.1) | *Green Chem.*, 2020, 22, 6540 |
| Cu NCs/Al_2_O_3_-10C | 0.5 M KHCO_3_ | 32 (-0.9)  43 (-1.0)  53.8 (-1.1) | -5.12 (-0.9)  -7.96 (-1.0)  -13.5 (-1.1) | *Angew. Chem*. 2021, 133, 25042. |
| Sb/CuO(V_O_) | 0.5 M KHCO_3_ | 43 (-1)  45 (-1.05)  46.2 (-1.1) | -5.2 (-1)  -5.2 (-1.05)  -6.8 (-1.1) | *SmartMat.* 2022, 3, 194 |
| MOF-derived Cu@Cu_x_O | 0.1 M KHCO_3_ | 20 (-0.78)  30 (-0.98)  38 (-1.18) | -4.4 (-0.78)  -7.5 (-0.98)  -11.4 (-1.18) | *J. Mater. Chem. A*, 2020, 8, 11117-11123 |
| CTF-B | 0.5 M KHCO_3_ | 25.3 (-1.38)  30.6 (-1.48) | -4.05 (-1.38)  -14.4 (-1.48) | *ACS Catal.* 2020, 10, 8, 4534 |
| Cu@BIF-144(Zn) | 0.5 M KHCO_3_ | 8 (-1.1)  12.9 (-1.5) | -0.3 (-1.1)  -1.2 (-1.5) | *Small*, 2023, 2305199 |
| Gd-CuO-e | 0.5 M KHCO_3_ | 38 (-1.0)  58.6 (-1.2) | -7 (-1.0)  -32.9 (-1.2) | *J. Mater. Chem. A*, 2024, 10.1039/d4ta05284f |
| Cu_2_O-Pd-Ag | 0.5 M KHCO_3_ | 37.5 (-1.0)  58 (-1.1) | -5 (-1.0)  -12.5 (-1.2) | *ACS Appl. Mater. Interfaces*, 2024, 16, 16243. |
| Cu NPs PSMIN | 0.5 M KHCO_3_ | 39 (-0.9) | -28 (-0.9) | *J. Mater. Chem. A,* 2024, 12, 11968 |
| Ag@BIF-104NSs(Cu) | 0.5 M KHCO_3_ | 21.43 (-1.1) | -20 (-0.9) | *Adv. Energy Mater.* 2023, 13, 2300088 |
| TPTA-Pz-COF | 0.5 M KHCO_3_ | 19.1 (-1.0) | -2.2 (-1.0) | *Angew. Chem. Int. Ed*. 2024, 63, e202404738 |
| Cu-[CF_2_]_n_-5-CP | 0.1 M CsI | 40 (-0.85)  47.5(-0.95)  48 (-1.05) | -5 (-0.85)  -13 (-0.95)  -17 (-1.05) | *Angew. Chem. Int. Ed.* 2024, 63, e202313796 |
| Cu-BmimNO_3_ | 0.1 M KHCO_3_ | 40 (-1.19) | -17.5 (-1.05) | *Angew. Chem. Int. Ed.* 2022, *61,* e202200039 |
| Cu/Cu_2+1_O/NC | 0.1 M KHCO_3_ | 17 (-0.9)  37 (-1)  47 (-1.05) | -3 (-0.9)  -8 (-1)  -13 (-1.05) | *Angew. Chem. Int. Ed.* 2024, e202413005 |
| Cu_1_Sn_1_ | 0.5 M KHCO_3_ | 85 (-1.0)  87 (-1.1) | 0.119 (-1.0) | *ACS Catal.*, 2021, 11, 11103. |

**Figure S13.** In-situ Raman spectra of CeCuO_x_ at different times at -0.93 V.

**Table S3.** Ce and Cu concentrations of CeCuO_x_ before and after test.

| Element/content | Ce | Cu |
| --- | --- | --- |
| Before test/μg | 61.37 | 188.73 |
| After test/μg | 22.40 | 115.00 |


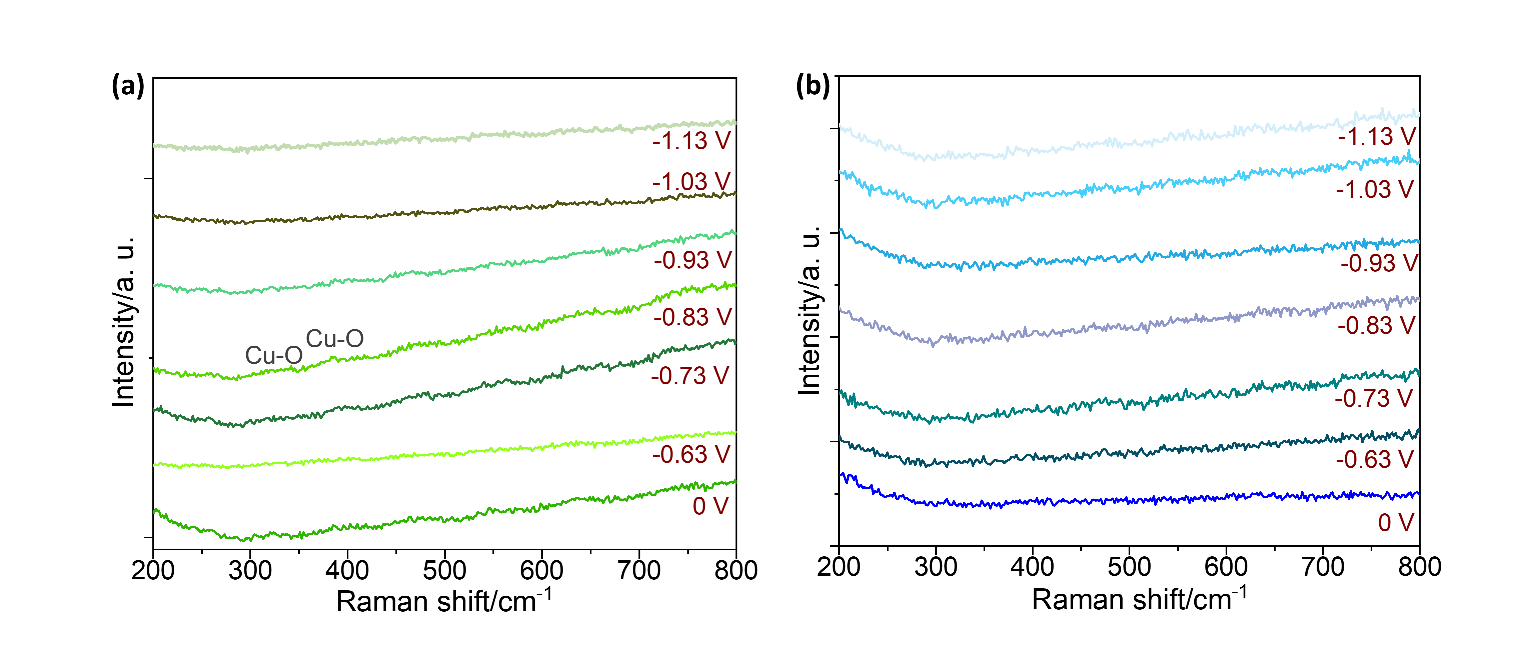
 **Figure S14.** In-situ Raman spectra of (a) Cu and (b) CuO_x_ measured at different potentials.

**
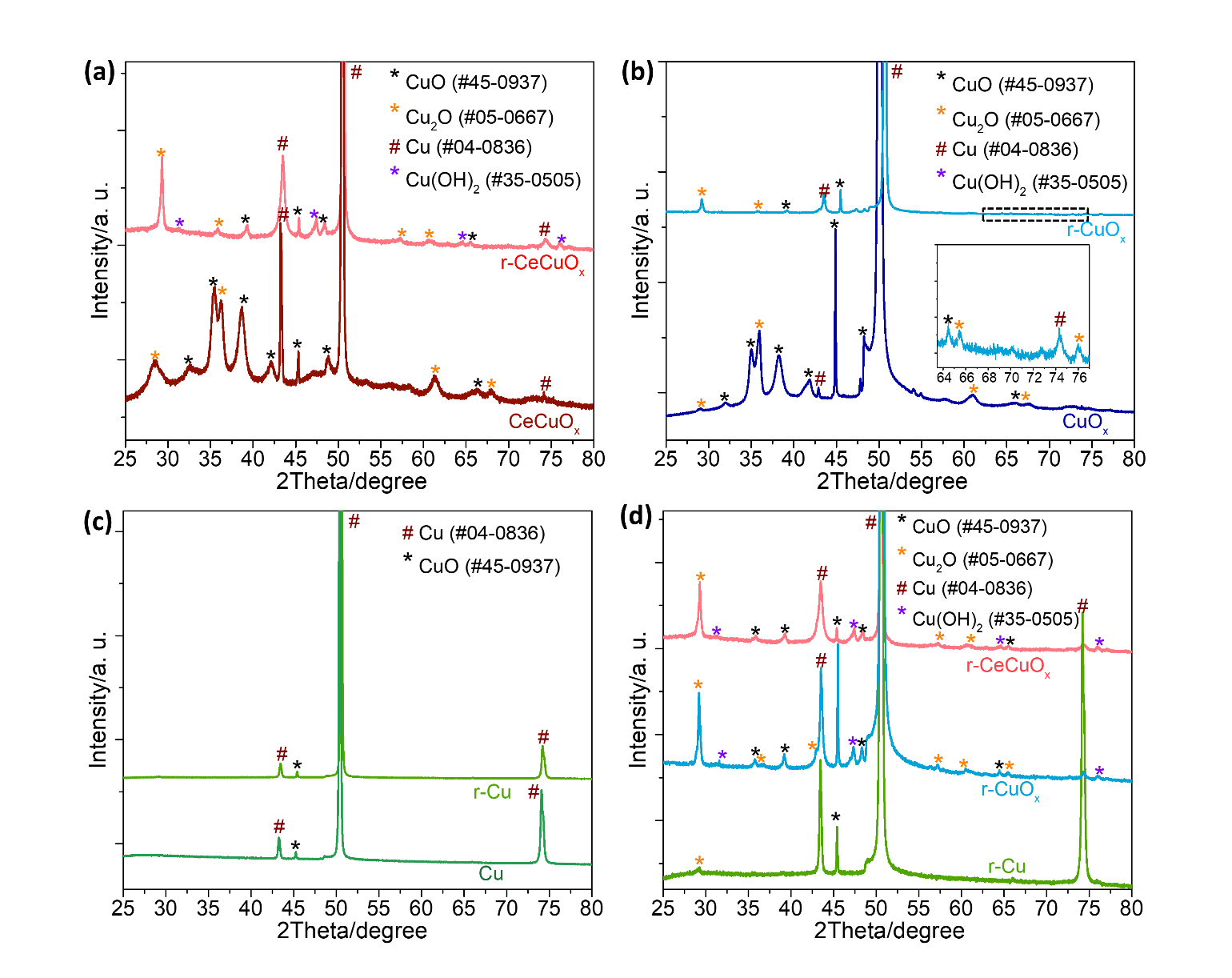
**

**Figure S15.** XRD patterns of (a) CeCuO_x_, (b) CuO_x_ and (c) Cu before and after 70-minute e-CO_2_RR test at -0.93 V. (d) Comparison of r-CeCuO_x_, r-CuO_x_ and r-Cu XRD patterns after e-CO_2_RR test at -0.93 V (1 h after test).


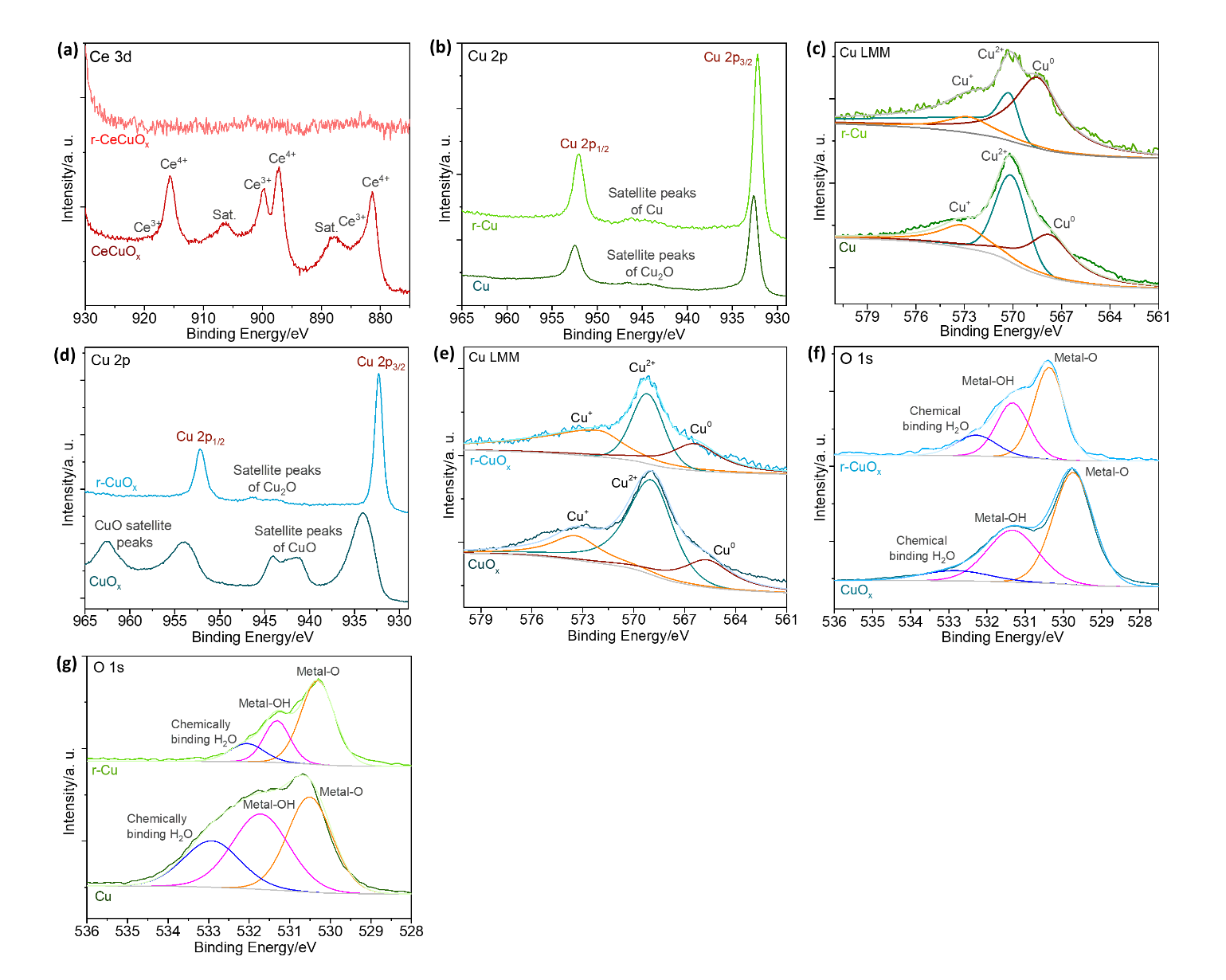


**Figure S16.** XPS spectra measured before and after e-CO_2_RR: (a) Ce 3d of CeCuO_x_, (b) Cu 2p and (c) Cu LMM of Cu, (d) Cu 2p and (e) Cu LMM spectra of CuO_x_, and O 1s spectra of (f) CuO_x_ and (g) Cu.


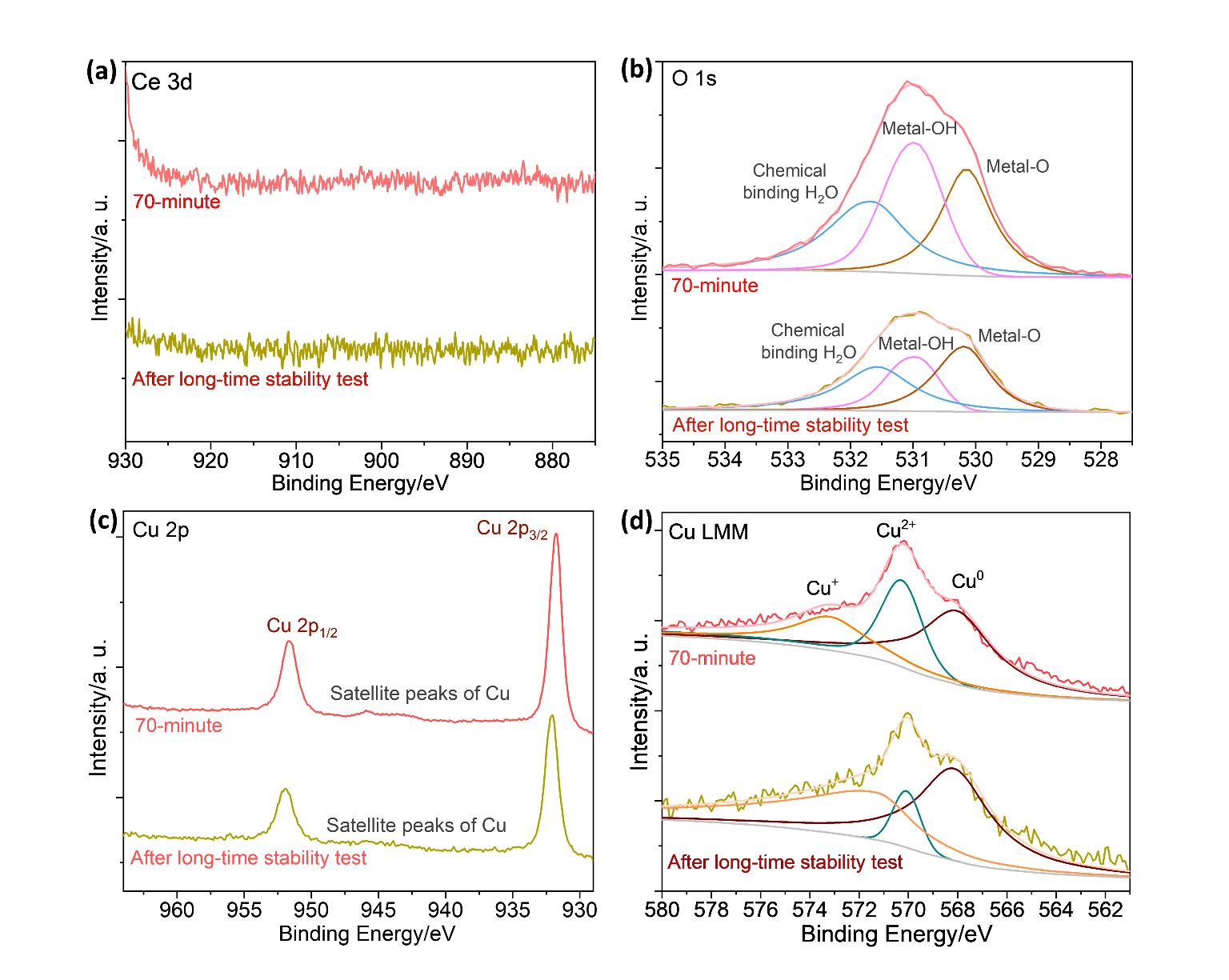


**Figure S17.** XPS spectra measured at 70-minute and 600-hour of CeCuO_x_ after e-CO_2_RR electrocatalysis: (a) Ce 3d, (b) O 1s, (c) Cu 2p and (d) Cu LMM.


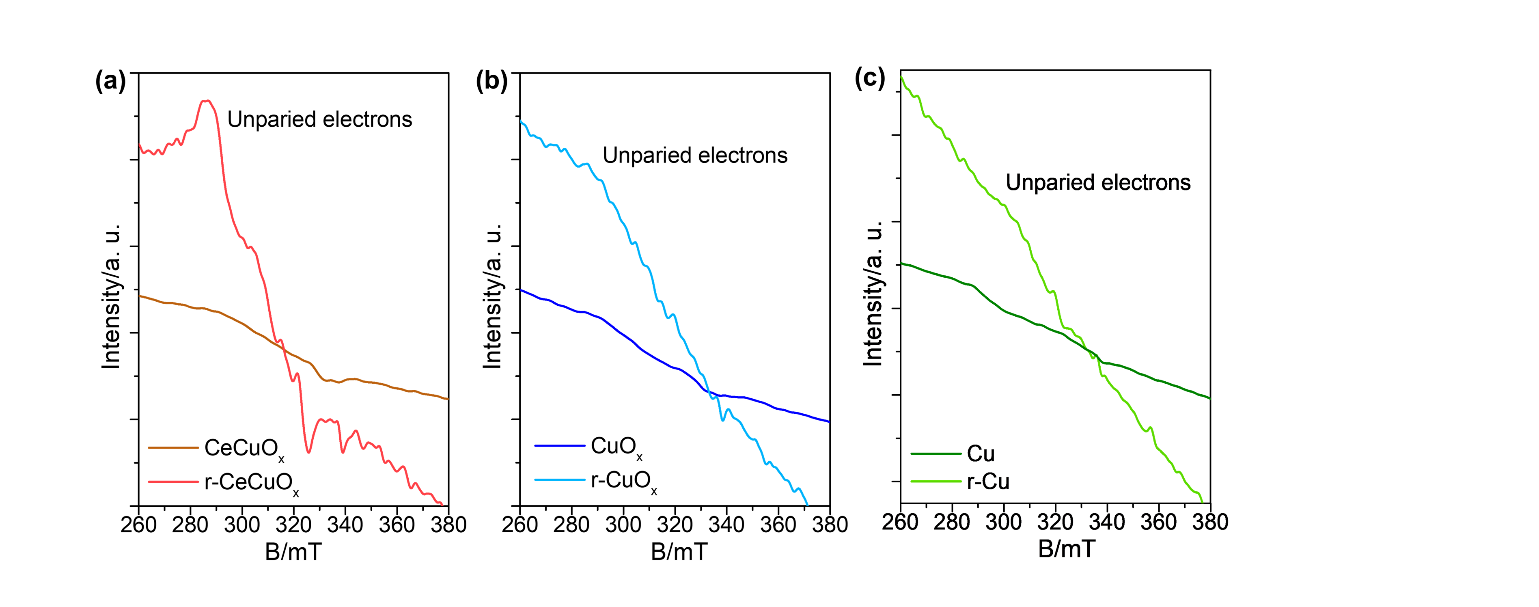


**Figure S18.** ESR spectra of CeCuO_x_, CuO_x_ and Cu before and after 70-minute e-CO_2_RR electrocatalysis.


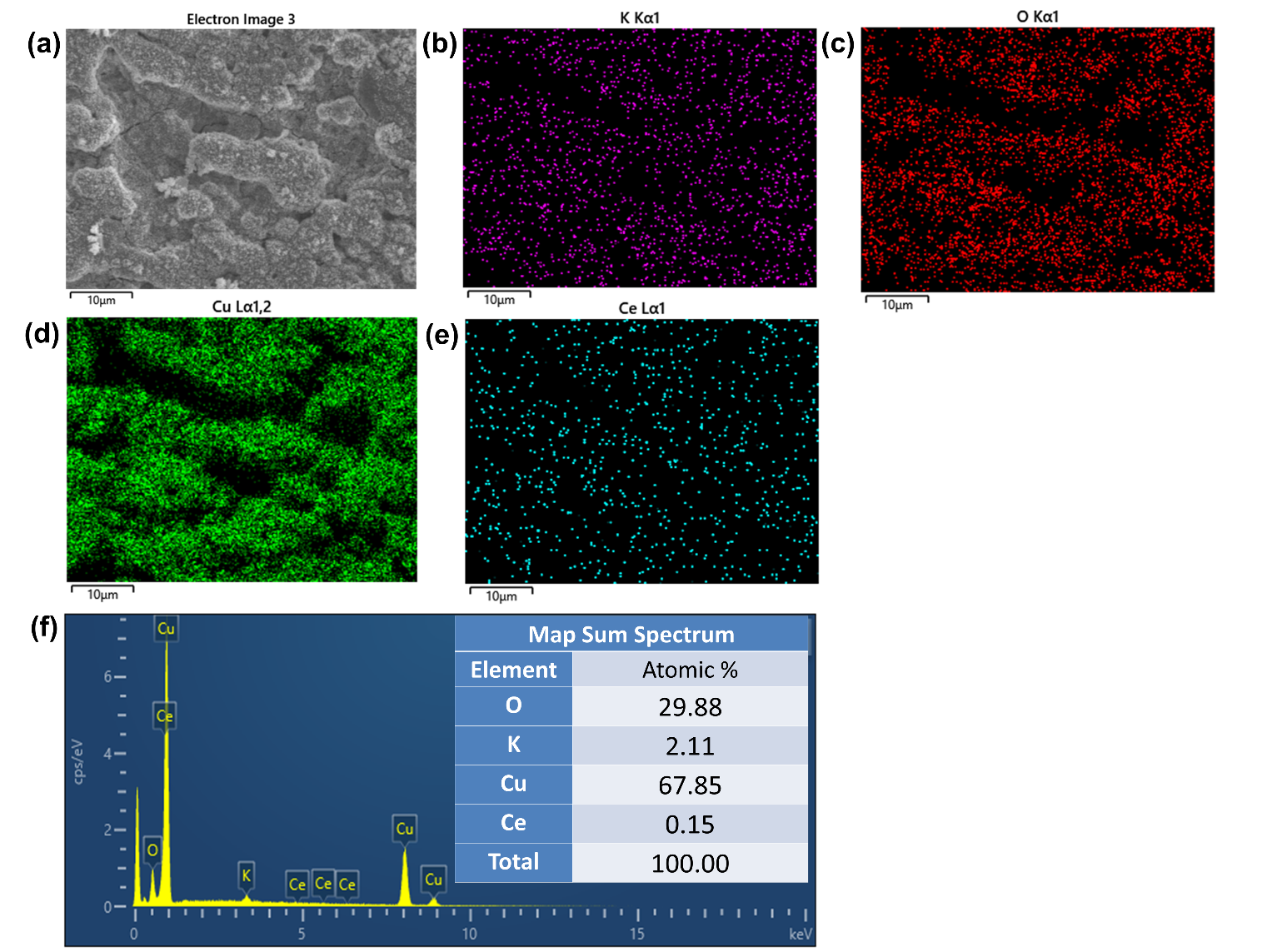


**Figure S19.** EDS element distribution and content of r-CeCuO_x_: (a) SEM image of selected area, (b) K, (c) O, (d) Cu and (e) Ce. (h) EDS spectrum with element content table.

**
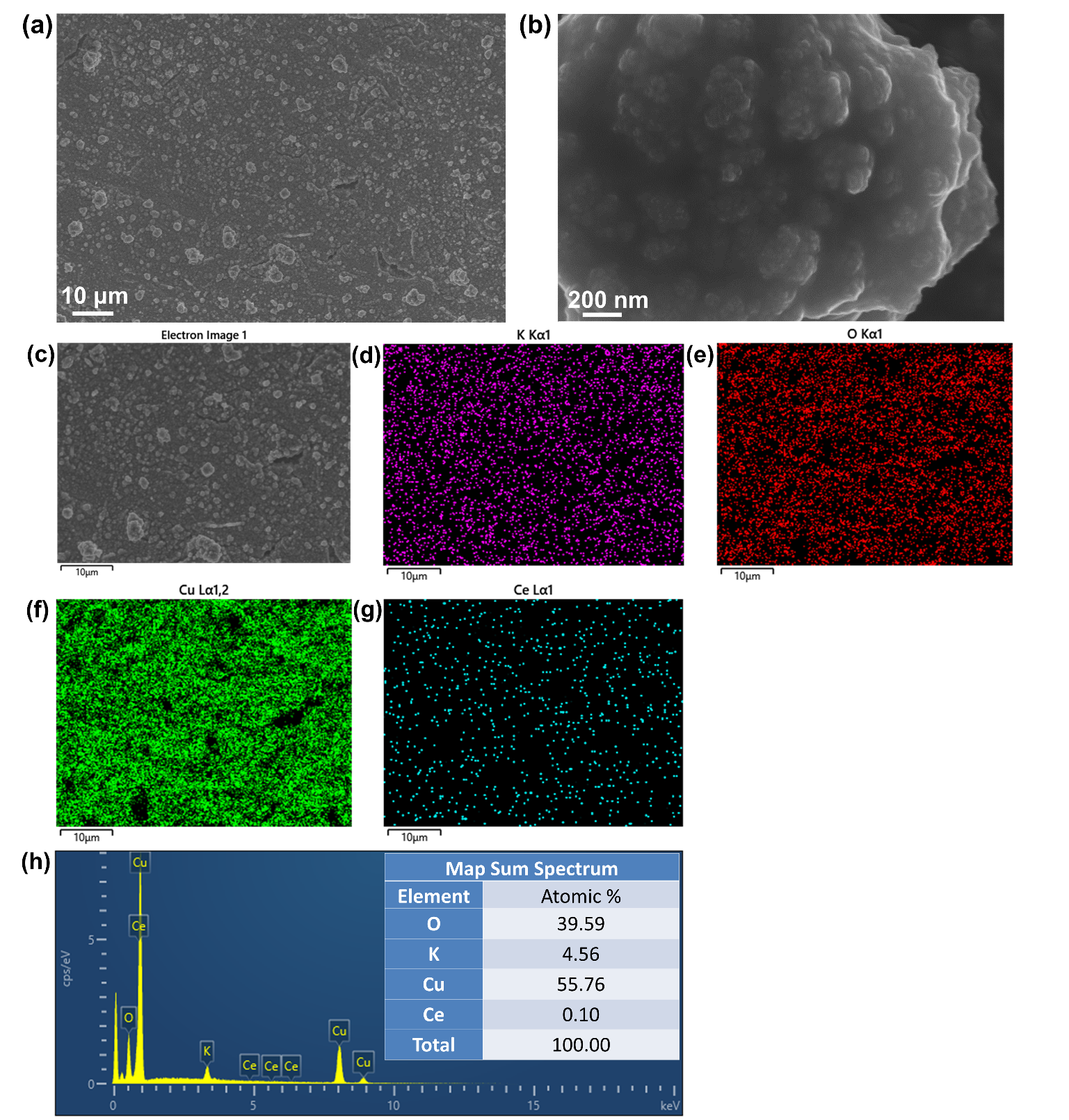
**

**Figure S20.** (a-b) SEM image of r-CuO_x_. EDS element distribution and content: (c) SEM image of selected area, (d) K, (e) O, (f) Cu and (g) Ce. (h) EDS spectrum with element content table.


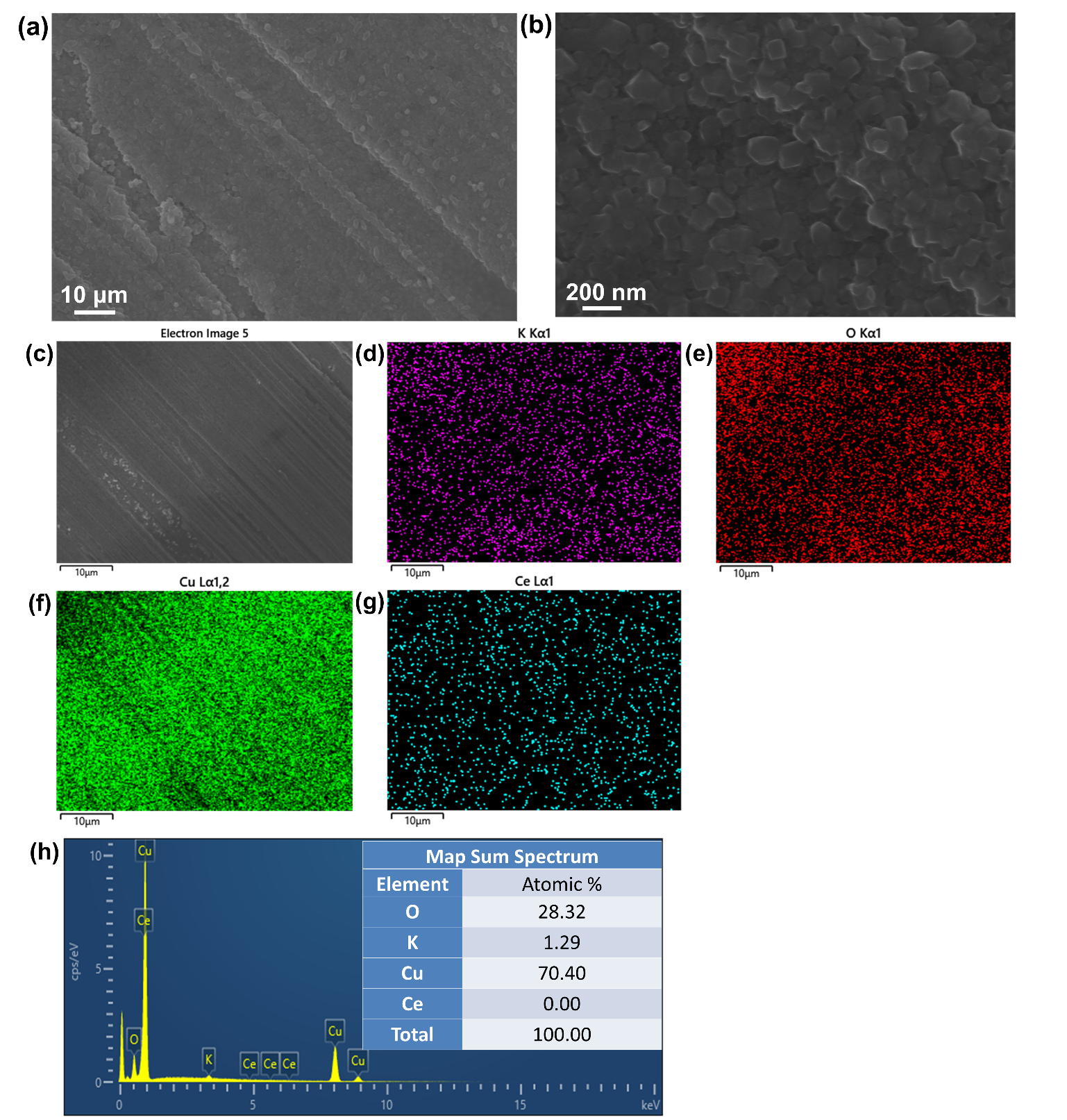


**Figure S21.** (a-b) SEM images of r-Cu. EDS element distribution and content: (c) SEM image of selected area, (d) K, (e) O, (f) Cu and (g) Ce. (h) EDS spectrum with element content table.

**
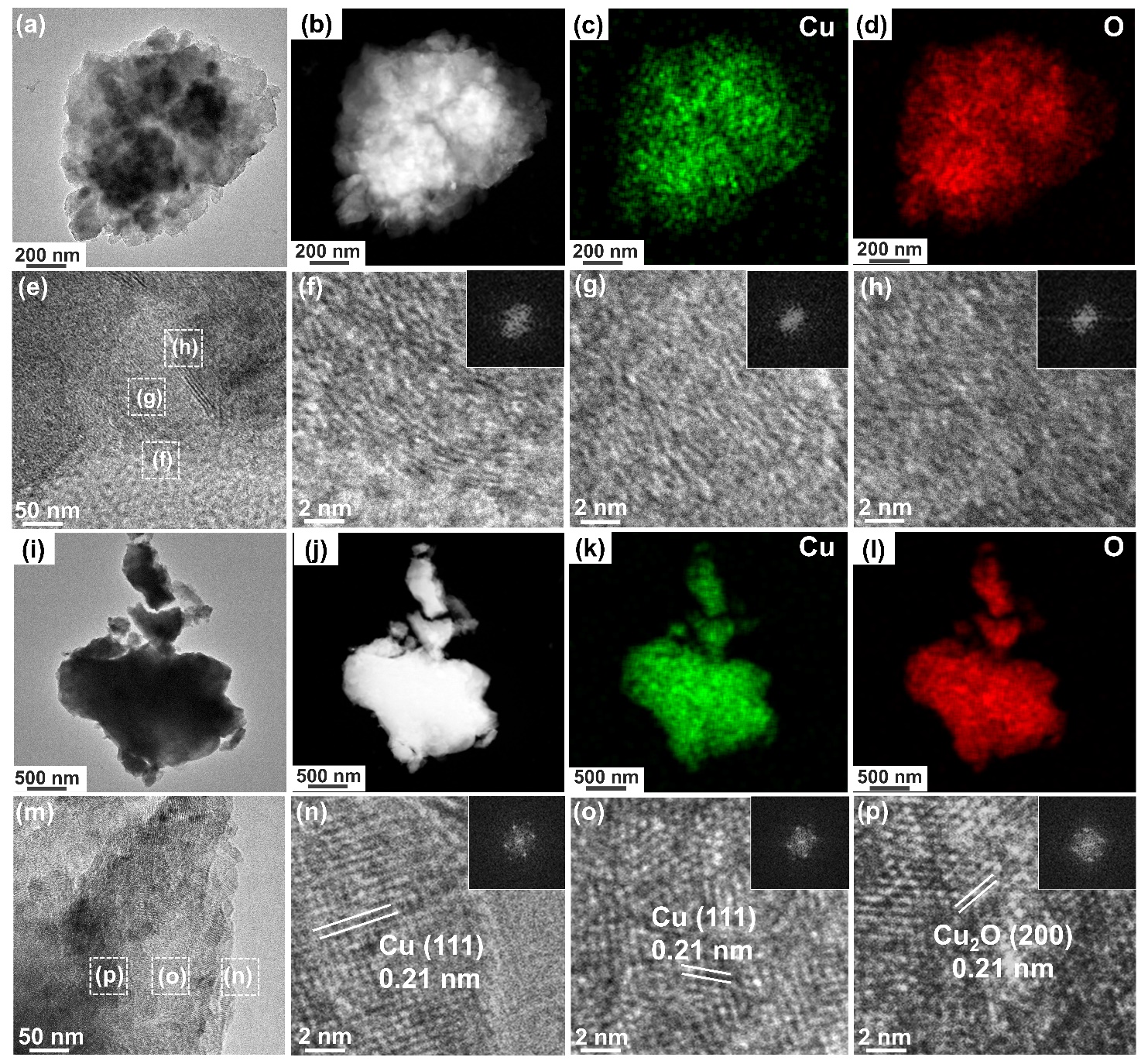
**

**Figure S22.** TEM images of r-CuO_x_ (a) and r-Cu (i). HAADF images of r-CuO_x_ (b) and r-Cu (j); EDS Cu and O mapping images of r-CuO_x_ (c-d) and r-Cu (k-l); HRTEM images of r-CuO_x_ (e) and r-Cu (m). High magnification of different positions in r-CuO_x_ (f-h) and r-Cu (n-p) (Inset: FFT image).

**Table S4.** XPS Cu^0^ versus Cu^+^, Cu^2+^ and Cu^+^+Cu^2+^ Auger peak area ratios of r-CeCuO_x_, r-CuO_x_ and r-Cu.

|  | Cu^0^/Cu^+^ | Cu^0^/Cu^2+^ | Cu^0^/(Cu^+^ +Cu^2+^) |
| --- | --- | --- | --- |
| r-Cu | 4.413 | 1.820 | 1.288 |
| r-CuO | 0.664 | 0.974 | 0.395 |
| r-CeCuO_x_ | 1.821 | 1.614 | 0.856 |

**Table S5.** XPS Cu^0^, Cu^+^ and Cu^2+^ Auger peak binding energy shifts of r-CuO_x_ and r-Cu compare with those of r-CeCuO_x_ (Binding energy shifts of r-CeCuO_x_ are set to 0 eV).

|  | r-CeCuO_x_ | r-CuO_x_ | r-Cu |
| --- | --- | --- | --- |
| Cu^0^ | 0 | -0.59 | +0.47 |
| Cu^+^ | 0 | -0.36 | -0.40 |
| Cu^2+^ | 0 | 0 | 0 |

The XPS Cu LMM spectra of r-CeCuO_x_ shows a lower ratio of Cu^0^/(Cu^+^+Cu^2+^) than those of r-CuO_x_, but higher than those of r-Cu (Figure 4b in the manuscript and Table S3). Notably, the XPS binding energies of Cu^0^ follows the trend: r-Cu > r-CeCuO_x_ > r-CuO_x_, while the XPS binding energies of Cu^+^ follows the trend: r-CeCuO_x_ > r-CuO_x_ > r-Cu (Figure 4b in the manuscript and Table S4), suggesting the electron density of Cu^0^ in r-CeCuO_x_ is higher than that in r-Cu, but lower than that of r-CuO_x_, while the Cu^+^ in r-CeCuO_x_ has lower electron density than those in r-CuO_x_ and r-Cu.

**Table S6.** XPS metal-O, metal-OH and chemical binding water binding energy shifts of r-CuO_x_ and r-Cu compare with those of r-CeCuO_x_ (Binding energies shifts of r-CeCuO_x_ are set to 0 eV).

|  | r-CeCuO_x_ | r-CuO_x_ | r-Cu |
| --- | --- | --- | --- |
| Metal-O | 0 | +0.26 | +0.18 |
| Metal-OH | 0 | +0.37 | +0.34 |
| Chemical binding water | 0 | +0.54 | +0.29 |

The XPS O 1s spectra show that the metal-O and metal-OH peaks of r-CeCuO_x_ show negative shifts compared with those of r-CuO_x_ and r-Cu (Figure 4c in the manuscript and Table S5), indicating the O atoms of r-CeCuO_x_ have higher electron density.^[2]^ Additionally, the O 1s peak of chemical binding water show negative shift, too, indicating the chemical binding water is negatively charged, so it shall be activated.

**Table S7.** CuO·3H_2_O and Cu_2_O phases in XRD patterns measured at 30^th^ minute after test.

|  | r-CeCuO_x_ | r-CuO_x_ | r-Cu |
| --- | --- | --- | --- |
| CuO·3H_2_O | (211), (014), (005) | (202), (211), (014), (005) | (202), (211), (005) |
| Cu_2_O | (220), (111), (200) | (111), (200) | (111) |
| *p*Cu(II) | 0.5 | 0.8 | 0.75 |

The index of Cu(II) compounds (*p*Cu(II)) (defined as the ratio between the number of CuO·3H_2_O peaks and number of all the oxidized Cu compounds diffraction peaks) is used to estimate the activity of the Cu species. The *p*Cu^2+^ value of r-CeCuO_x_ (*p*Cu(II) = 0.5) is lower than that of r-CuO_x_ (*p*Cu(II) = 0.8) and r-Cu (*p*Cu(II) = 0.75) (Table S6), indicating the Cu (II) species generation shall be more favorable.


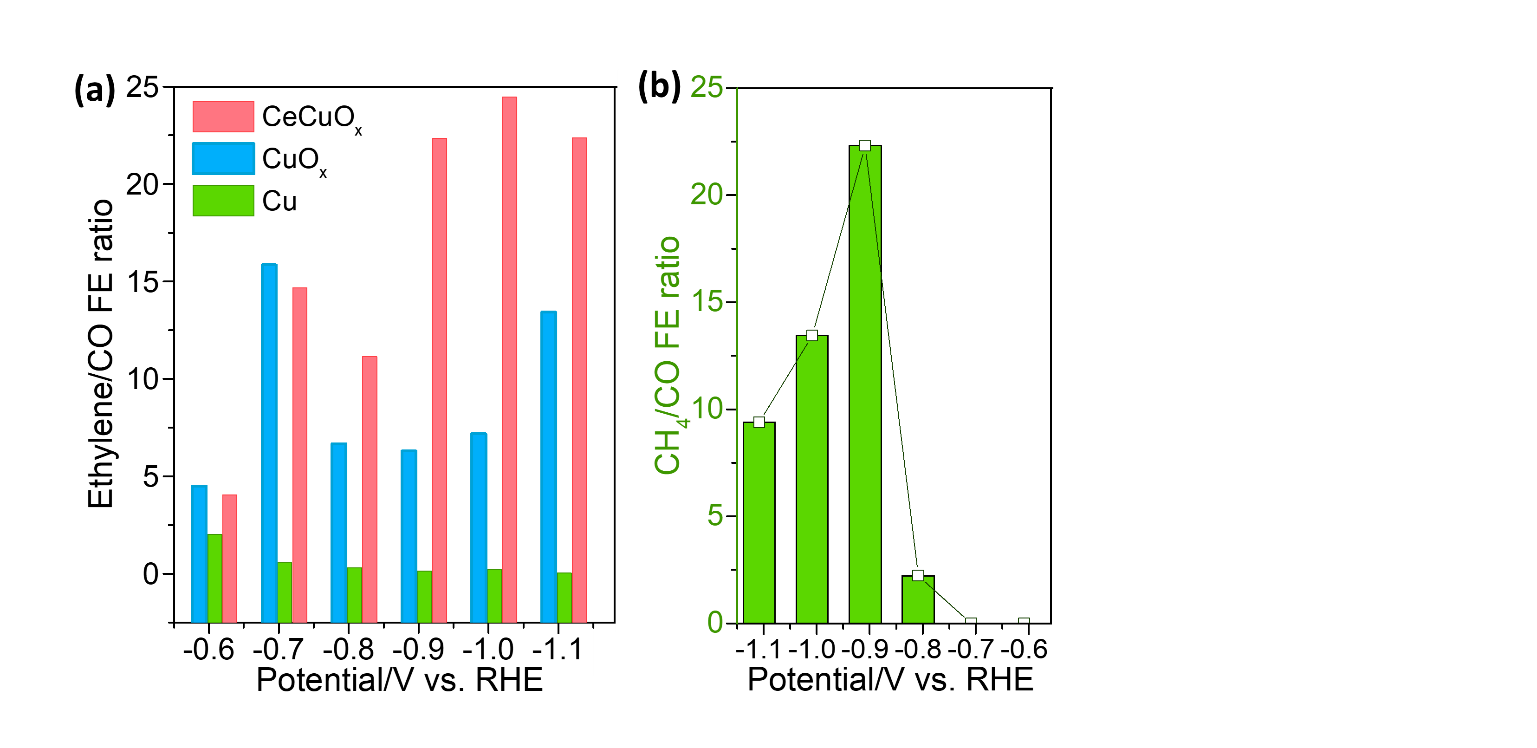


**Figure S23.** (a) Potential-dependent ethylene/CO ratios of r-Cu, r-CuO_x_ and r-CeCuO_x_ and (b) CH_4_/CO ratios of r-Cu.


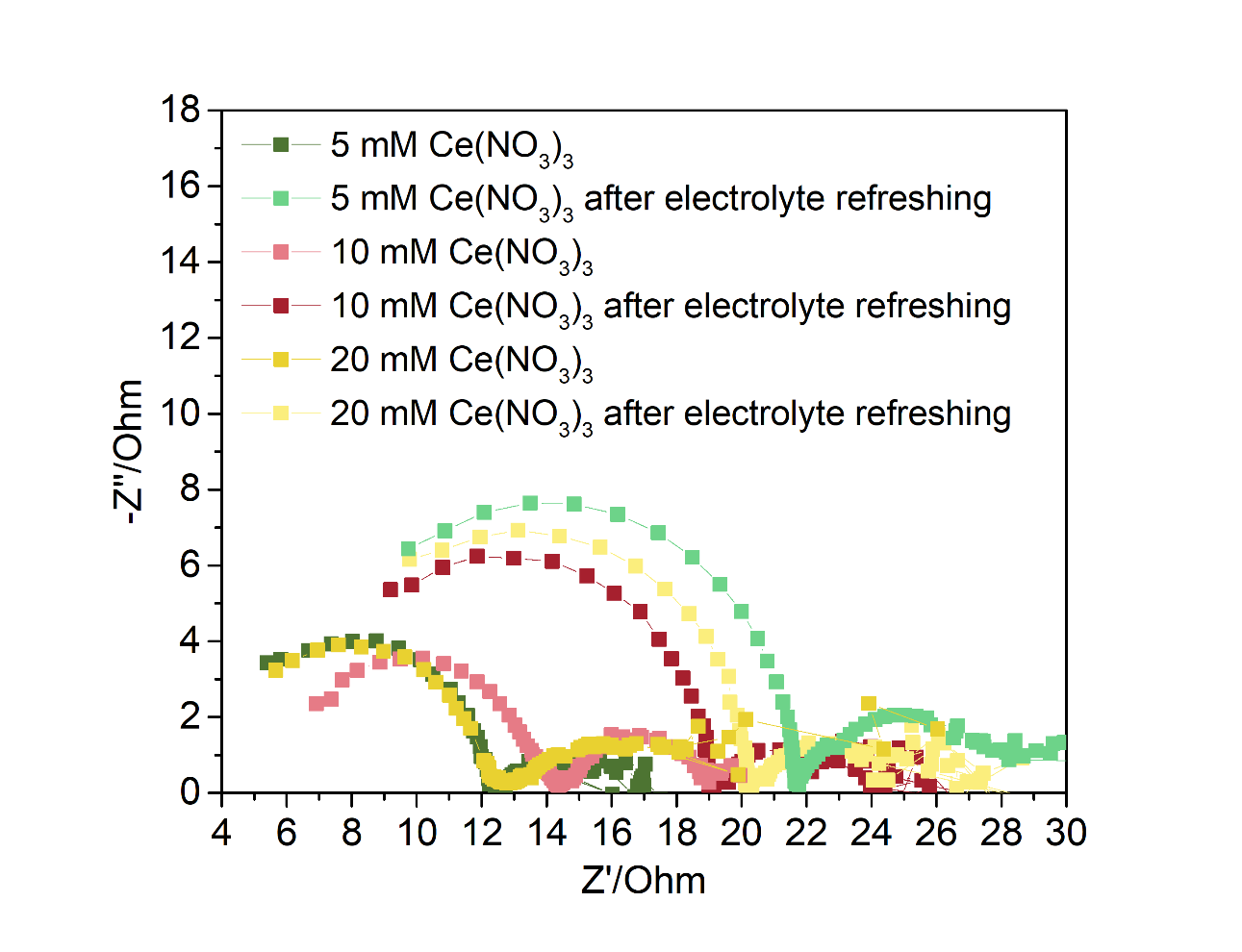


**Figure S24.** Nyquist plots of CeCuO_x_ prepare by using different Ce(NO_3_)_3_ concentrations as Ce sources before and after electrolyte refreshing at post -0.93 V test.


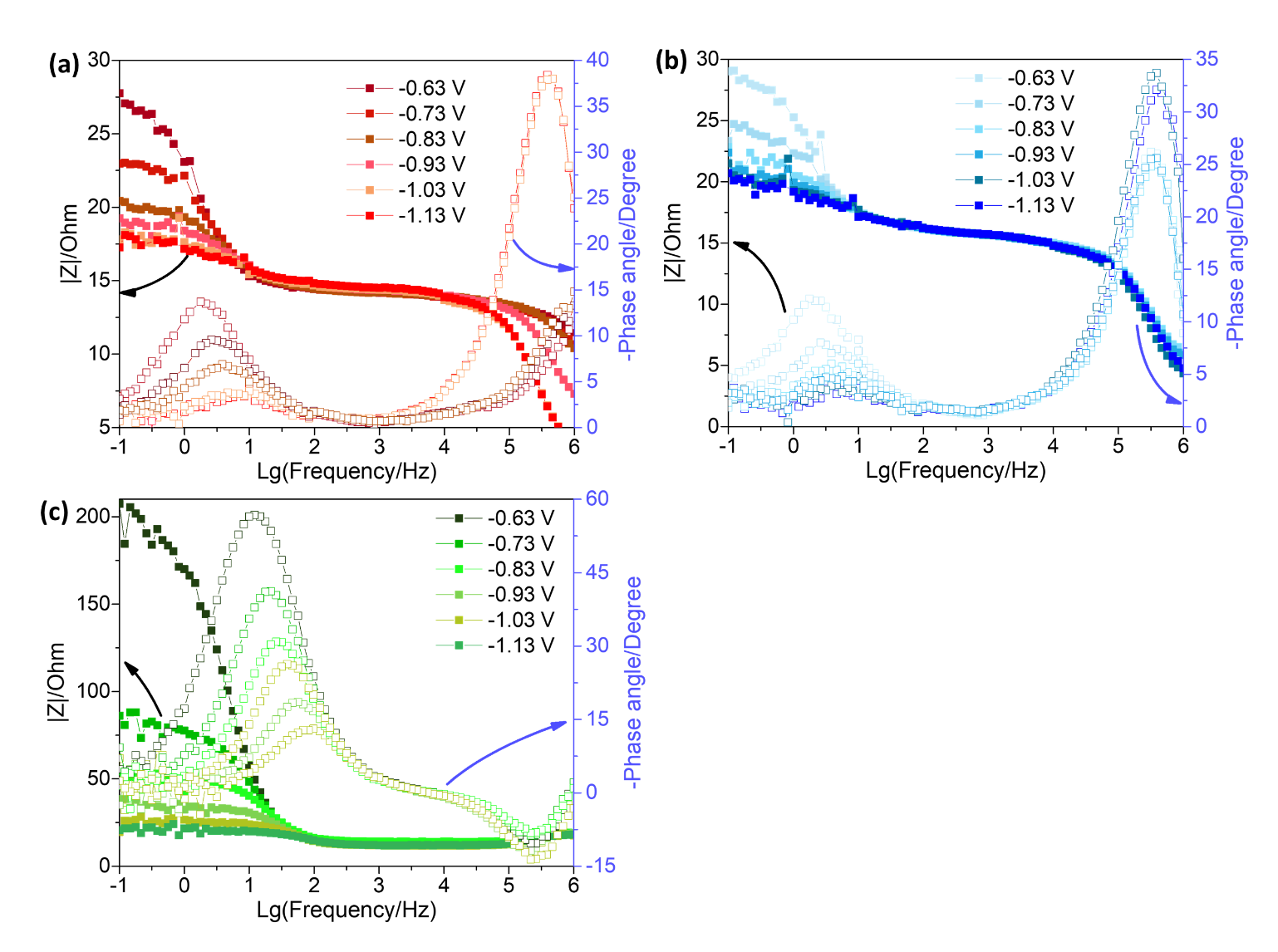


**Figure S25.** Bode plots of (a) r-CeCuO_x_, (b) r-CuO_x,_ and (c) r-Cu.


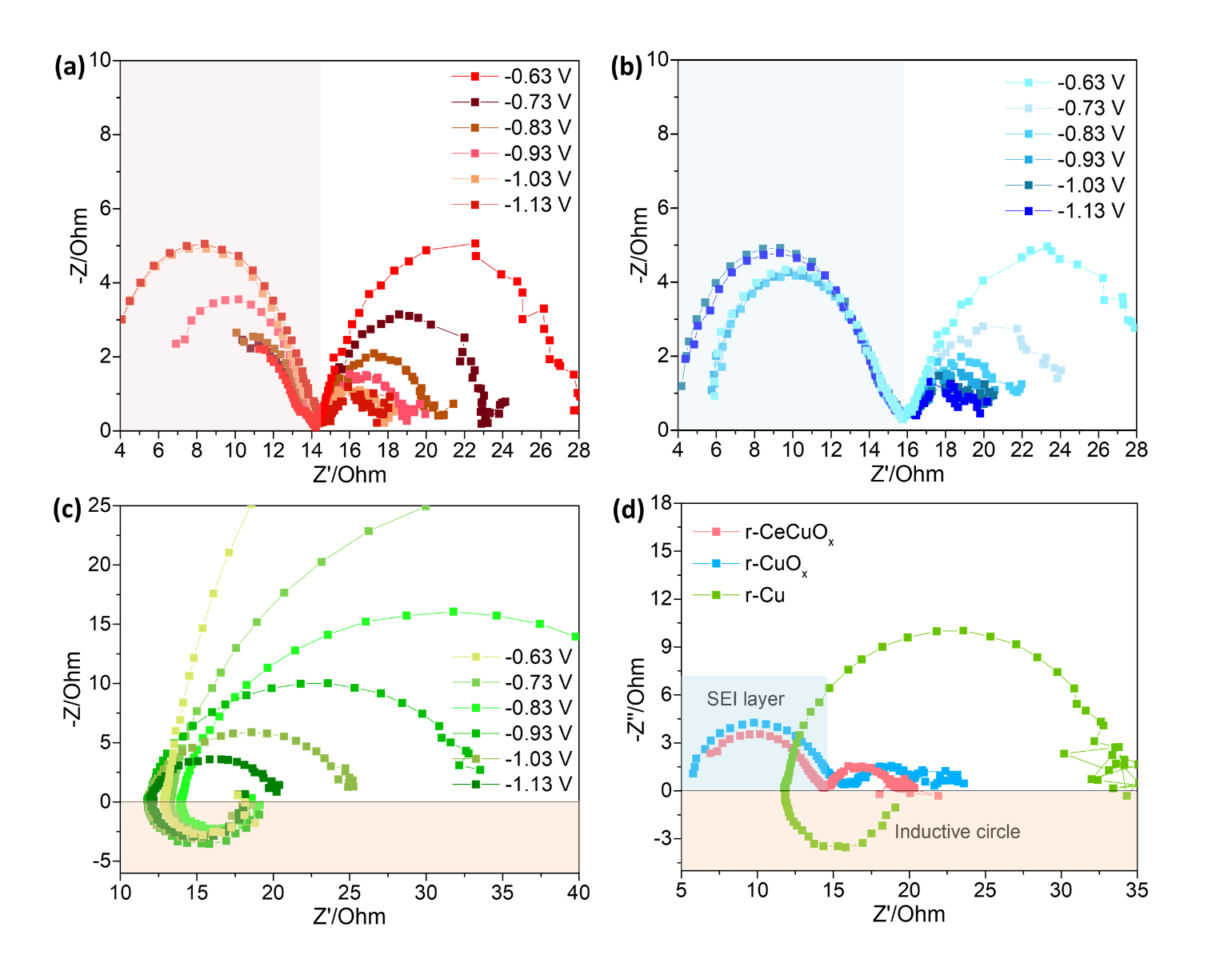


**Figure S26.** Nyquist plots of electrocatalysts after e-CO_2_RR tests: (a) CeCuO_x_, (b) CuO_x_ and (c) Cu. (d) Electronic circuit used to fit.


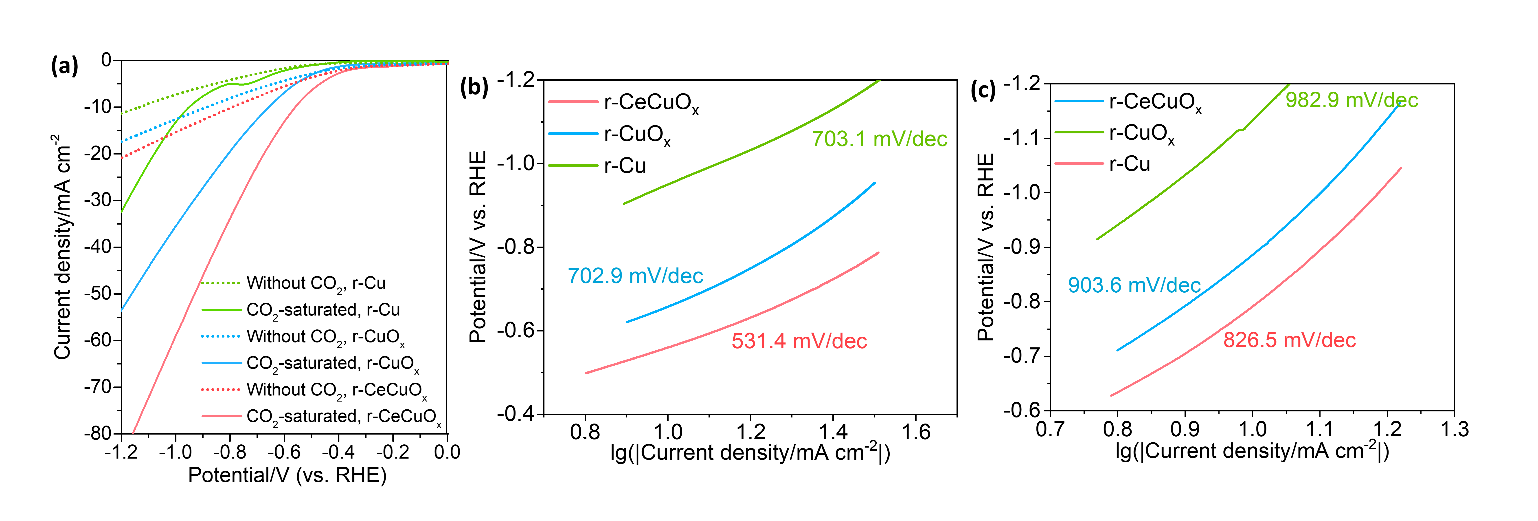


**Figure S27.** (a) LSV curves measure with and without CO_2_. Tafel plots of r-CeCuO_x_, r-CuO_x_ and r-Cu with (b) and (c) without CO_2_.

**Figure S28.** CV curves of r-CeCuO_x_, r-CuO_x_ and r-Cu in CO_2_-saturated 0.5 M KHCO_3_ after e-CO_2_RR test at -0.93 V.

**Figure S29.** ESR spectra of electrolyte with 0.01 M DMPO using r-Cu, r-CuO_x_ and r-CeCuO_x_ as electrocatalysts after 70-minute e-CO_2_RR test.


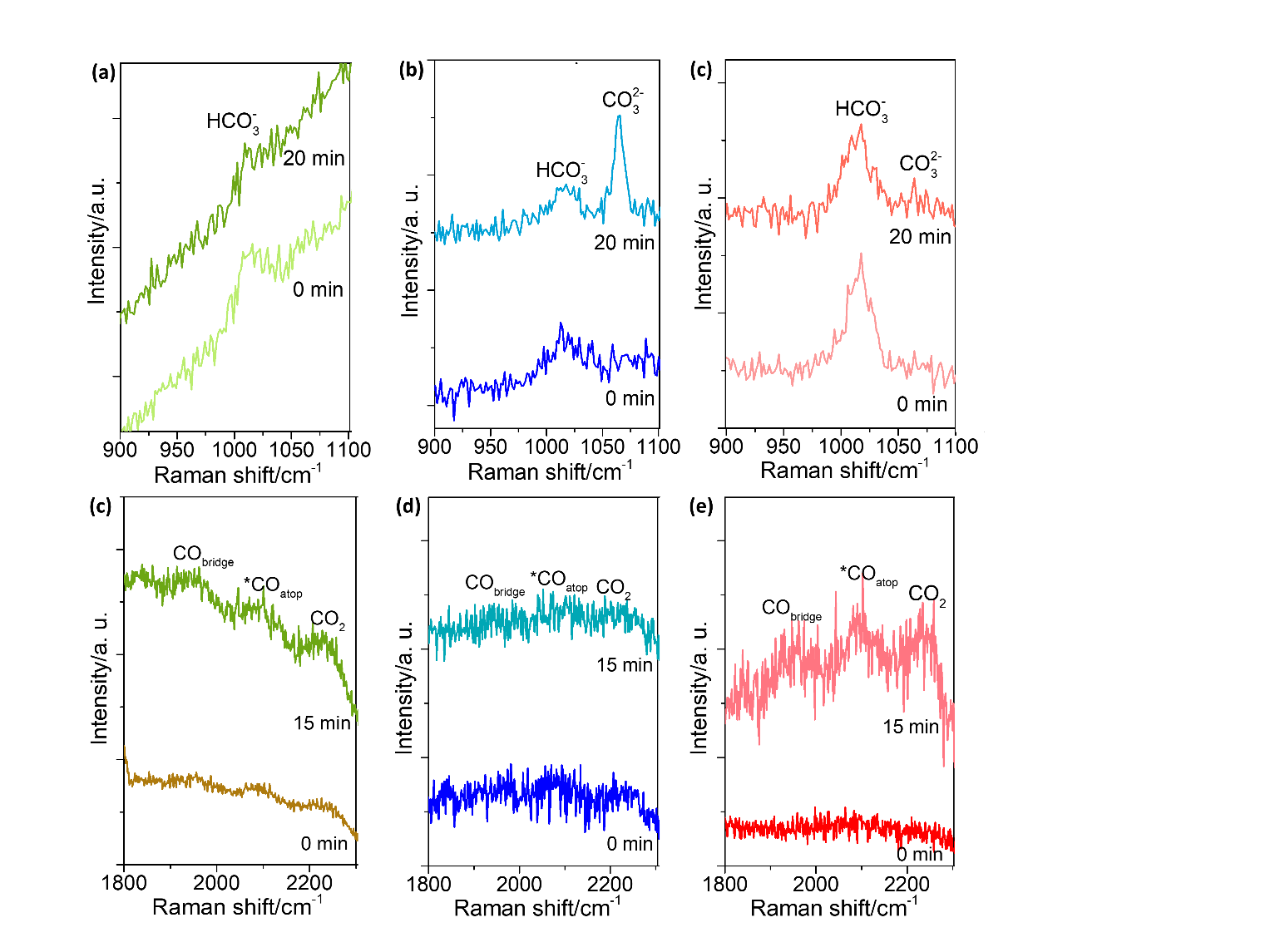


**Figure S30.** In-situ Raman spectra measured at -0.93 V for different reaction time: (a) r-Cu, (b) r-CuO_x_ and (c) r-CeCuO_x_ at 900-1100 cm^-1^ at 0 and 20^th^ minute; at 1800-2200 cm^-1^ at 0 and 15^th^ minute: (d) r-Cu, (e) r-CuO_x_ and (f) r-CeCuO_x_.

**References**

[1] L. Majidi, A. Ahmadiparidari, N. Shan, S. N. Misal, K. Kumar, Z. Huang, S. Rastegar, Z. Hemmat, X. Zou, P. Zapol, J. Cabana, L. A. Curtiss, A. Salehi-Khojin, *Adv. Mater.* **2021**, *33*, e2004393.

[2] L. Ley, R. A. Pollak, F. R. McFeely, S. P. Kowalczyk, D. A. Shirley, *Phys. Rev. B* **1974**, *9*, 600-621.
